# Cyclodextrins as Modulators of Regulated Cell Death: Implications for Immunometabolism and Therapeutic Innovation

**DOI:** 10.3390/pharmaceutics18030306

**Published:** 2026-02-28

**Authors:** Diana-Maria Trasca, Andreea Gabriela Mocanu, Ion Dorin Pluta, Cristina Popescu, George Alin Stoica, Renata Maria Varut, Denisa Preoteasa, Ștefănița Bianca Vintilescu, Mioara Desdemona Stepan, Cristina Elena Singer, Denisa Floriana Vasilica Pirscoveanu

**Affiliations:** 1 Department of Internal Medicine, University of Medicine and Pharmacy of Craiova, 200349 Craiova, Romania; diana.trasca@umfcv.ro; 2 Department of Pharmaceutical Technology, University of Medicine and Pharmacy of Craiova, 200349 Craiova, Romania; gabriela.mocanu@umfcv.ro; 3 Faculty of Medical and Behavioral Sciences, Constantin Brâncuși University of Târgu Jiu, 210185 Târgu Jiu, Romania; dorin.pluta@e-ucb.ro; 4 ENT County Hospital Craiova, Discipline of Anatomy, Department of Anatomy, University of Medicine and Pharmacy, 200349 Craiova, Romania; cristina.popescu@umfcv.ro; 5 Department of Pediatric Surgery, Faculty of Medicine, University of Medicine and Pharmacy of Craiova, 200349 Craiova, Romania; 6 Research Methodology Department, Faculty of Pharmacy, University of Medicine and Pharmacy of Craiova, 200349 Craiova, Romania; 7 Vâlcea County Emergency Hospital, 240562 Râmnicu Vâlcea, Romania; denisa_chiosa@yahoo.com; 8 Department of Mother and Baby, University of Medicine and Pharmacy of Craiova, 200349 Craiova, Romania; bianca.vintilescu@umfcv.ro (Ș.B.V.); desdemona.stepan@umfcv.ro (M.D.S.); cristina.singer@umfcv.ro (C.E.S.); 9 Department of Neurology, Faculty of Medicine, University of Medicine and Pharmacy of Craiova, 200349 Craiova, Romania; denisa.pirscoveanu@umfcv.ro

**Keywords:** cyclodextrins, hydroxypropyl-β-cyclodextrin, methyl-β-cyclodextrin, regulated cell death, immunometabolism, cholesterol homeostasis, lysosome, macrophage polarization, drug delivery, translational pharmaceutics

## Abstract

This review critically examines how cyclodextrins modulate regulated cell death pathways and the implications for immunometabolism and therapeutic translation. Increasing evidence, however, indicates that cyclodextrins exert intrinsic biological activity by modulating cellular lipid homeostasis, membrane organization, and intracellular trafficking. In recent years, these properties have positioned cyclodextrins as unexpected regulators of regulated cell death (RCD) pathways, with broad implications for immunometabolism and therapeutic innovation. This review provides a comprehensive overview of the mechanisms by which native and chemically modified cyclodextrins influence major forms of regulated cell death, including apoptosis, autophagy-dependent cell death, pyroptosis, ferroptosis, and necroptosis. Particular attention is given to cholesterol sequestration, lipid raft disruption, lysosomal cholesterol mobilization, and transcriptional reprogramming via pathways such as TFEB (transcription factor EB) and AMPK (AMP-activated protein kinase), which collectively shape cell fate decisions. We further examine how cyclodextrin-mediated modulation of RCD intersects with immune metabolism, especially macrophage polarization and inflammasome activity, thereby influencing inflammatory responses and disease progression. Translational implications are discussed across diverse pathological contexts, including cancer, cardiovascular diseases, neurodegenerative disorders, inflammatory and autoimmune conditions, infectious diseases, and lysosomal storage disorders. Finally, emerging cyclodextrin-based delivery platforms, ranging from inclusion complexes to nanoparticles and polymeric systems, are evaluated with respect to their ability to achieve targeted modulation of cell death while minimizing off-target toxicity. Importantly, we critically discuss dose-dependent cytotoxicity, sterol depletion–related adverse effects, and formulation-dependent variability, which currently limit the clinical translation of cyclodextrin-mediated cell death modulation. By integrating mechanistic insights with pharmaceutical formulation strategies, this review delineates key challenges and opportunities for the rational design of cyclodextrin-based therapeutics. Overall, this review highlights cyclodextrins as bioactive modulators rather than inert carriers, underscoring their potential to inspire novel pharmacological strategies that integrate drug delivery, immunometabolism, and regulated cell death.

## 1. Introduction

Regulated cell death (RCD) encompasses a spectrum of genetically controlled pathways through which cells undergo demise in a programmed manner. Distinct forms of RCD, including apoptosis, autophagy-dependent cell death, pyroptosis, ferroptosis, and necroptosis, play crucial roles in development, homeostasis, and disease. Dysregulation of these death pathways is implicated in numerous pathologies, from cancer and neurodegeneration to inflammatory and cardiovascular diseases [1,2,3]. Consequently, there is intense interest in pharmacologically modulating RCD pathways for therapeutic benefit. In recent years, cyclodextrins (CDs), a family of cyclic oligosaccharides traditionally used as pharmaceutical excipients, have emerged as unexpected modulators of cell death processes. Cyclodextrins are toroidal molecules (typically composed of 6–8 glucopyranose units in α-, β-, or γ-CD) with a hydrophilic exterior and hydrophobic cavity, enabling them to encapsulate lipophilic molecules. This unique inclusion ability, especially pronounced in β-cyclodextrin and its derivatives, underlies many of the biological effects of CDs. By binding cholesterol and other lipids, CDs can alter membrane composition, intracellular lipid trafficking, and signaling cascades. Such effects have placed cyclodextrins at the intersection of cell death regulation and cellular metabolism [4,5,6,7].

Early observations that methyl-β-cyclodextrin (MβCD) could extract cholesterol from plasma membranes hinted at a potential to influence apoptosis and other death pathways by disrupting lipid raft-dependent signaling. Subsequent studies revealed that hydroxypropyl-β-cyclodextrin (HPβCD), a widely used derivative, not only mobilizes cholesterol from lysosomes but can also trigger cell death in certain contexts or alleviate pathological cell death in others. The pleiotropic effects of cyclodextrins on cell viability have spurred research into their mechanism of action and therapeutic applications. Notably, cyclodextrin-based interventions are being explored across a broad range of diseases: in oncology as chemosensitizers or drug carriers, in atherosclerosis as anti-atherogenic agents, in neurodegenerative and lysosomal storage disorders as cholesterol-clearing drugs, and in inflammatory and infectious diseases as modulators of immune cell function [7,8,9].

This review provides a comprehensive examination of how cyclodextrins modulate various regulated cell death pathways and the implications for immunometabolism and therapy. We first outline the mechanistic impact of cyclodextrins on the major RCD modalities, apoptosis, autophagy (and autophagy-dependent cell death), pyroptosis, ferroptosis, and necroptosis, highlighting examples of cyclodextrin-based systems (native or chemically modified) that influence these processes. We then discuss how these mechanistic insights translate to disease contexts, surveying the role of cyclodextrin-mediated RCD modulation in cancer; cardiovascular diseases; neurological disorders; inflammatory conditions; infectious diseases; and lysosomal storage disorders. A dedicated section is included on the immunometabolic effects of cyclodextrins, especially their ability to reprogram macrophage polarization between pro-inflammatory (M1) and anti-inflammatory (M2) states, linking cholesterol handling to innate immune activation. We also describe the emerging delivery platforms utilizing cyclodextrins, from inclusion complexes to nanoparticles and polymers, that enable targeted modulation of cell death pathways while aiming to minimize off-target toxicity.

Key concepts are summarized to provide a clear and structured overview of the field. Different cyclodextrin types and their derivatives are correlated with the RCD pathways they modulate, together with the underlying mechanisms involved. In addition, cyclodextrin-based interventions are discussed in relation to specific diseases from the perspective of cell death modulation, illustrating how these systems influence pathological outcomes through processes such as apoptosis, autophagy, and other forms of RCD. Finally, the translational dimension is highlighted by addressing the current stage of development of cyclodextrin-based therapeutic strategies, including clinically investigated CD formulations and emerging advanced delivery platforms. While several recent reviews have addressed cyclodextrins as drug delivery vehicles or cholesterol-modulating agents, none have systematically integrated regulated cell death pathways with immunometabolic reprogramming and pharmaceutical formulation strategies. In particular, the context-dependent duality of cyclodextrins as cytoprotective versus cytotoxic agents remains underexplored from a translational pharmaceutics perspective. In total, this review underscores that cyclodextrins, beyond their pharmaceutical carrier role, are bioactive modulators of cell death and immune metabolism. Leveraging these properties could inspire innovative therapies for diverse pathologies, while careful attention to safety (such as off-target cytotoxicity or sterol depletion in normal tissues) will be crucial. In the following sections, we delve into each RCD pathway and its intersection with cyclodextrin biology, setting the stage for understanding disease-specific applications and future directions.

The central premise of this review is that CDs should be regarded as bioactive modulators of regulated cell death rather than inert drug carriers. Their primary mechanism, cholesterol redistribution, initiates a cascade of lysosomal, metabolic, and inflammatory signaling events that determine context-dependent cell fate and therapeutic outcomes.

## 2. Cyclodextrins and Regulated Cell Death: Mechanistic Overview

**Figure 1:**
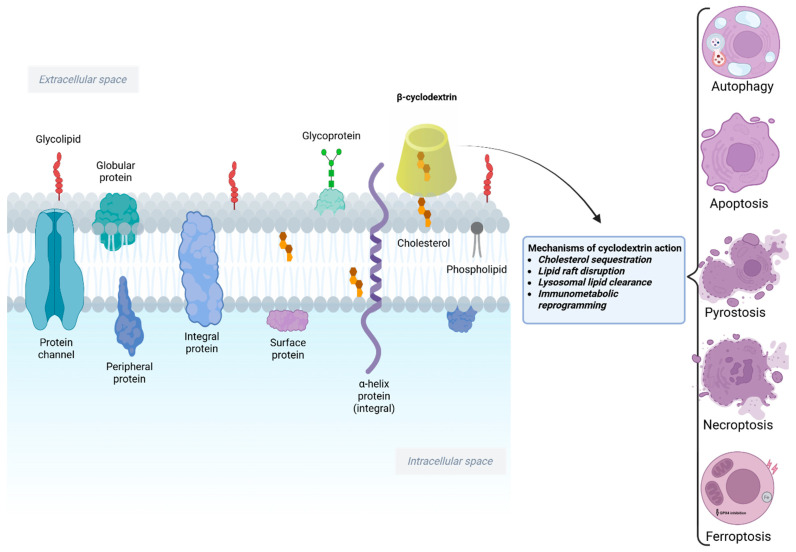
Cyclodextrin-mediated modulation of regulated cell death pathways. β-Cyclodextrin acts as a central modulator of regulated cell death by sequestering membrane cholesterol, disrupting lipid rafts, promoting lysosomal lipid clearance, and inducing immunometabolic reprogramming. Through these interconnected mechanisms, cyclodextrins influence apoptosis, autophagy, ferroptosis, pyroptosis, and necroptosis across diverse pathological contexts. Created in BioRender. Varut, M. (2026) https://BioRender.com/ygjxqua (accessed on 14 January 2026).

Regulated cell death (RCD) pathways play essential roles in tissue homeostasis, host defense, and disease pathogenesis. Beyond their classical use as pharmaceutical excipients, cyclodextrins have emerged as active modulators of multiple RCD modalities through their ability to sequester cholesterol, remodel cellular membranes, influence lysosomal function, and reprogram cellular metabolism. These interconnected effects place cyclodextrins at the crossroads of signaling pathways governing cell survival and death (Figure 1) [10,11]. To distinguish the intrinsic biological activity of CDs from their classical role as pharmaceutical excipients, several criteria should be considered. First, CD-induced cellular responses must be observed in the absence of a pharmacological cargo, demonstrating direct bioactivity. Second, these effects should involve modulation of key signaling pathways linked to regulated cell death and immunometabolism, such as TFEB activation, AMPK signaling, lysosomal functional changes, or inflammasome attenuation. Third, the biological outcome should display a concentration-dependent profile, with low or moderate doses promoting adaptive responses (e.g., restoration of autophagic flux and metabolic reprogramming), whereas higher concentrations induce cytotoxicity through excessive membrane lipid extraction and lysosomal destabilization. Together, these features support the classification of CDs as active modulators of cell fate rather than inert delivery vehicles.

### 2.1. Primary Cholesterol Depletion as the Initiating Event Versus Downstream Signaling Responses

The biological effects of cyclodextrins follow a hierarchical sequence in which cholesterol depletion represents the primary initiating event, while the activation of intracellular signaling pathways constitutes a secondary adaptive response. By directly extracting cholesterol from the plasma membrane and endolysosomal compartments, cyclodextrins remodel membrane microdomains, alter lipid-raft-dependent receptor organization, and reduce lysosomal lipid burden. These primary biophysical changes subsequently trigger downstream signaling processes, including TFEB activation, AMPK-dependent metabolic reprogramming, restoration of autophagic flux, and attenuation of NLRP3 inflammasome activity. Thus, cholesterol redistribution should be regarded as the upstream driver of cyclodextrin bioactivity, whereas the observed metabolic, inflammatory, and cell-death-related outcomes reflect context-dependent downstream responses that ultimately determine cell fate (Figure 1). Cholesterol depletion represents the primary trigger, whereas TFEB/AMPK activation, autophagy modulation, and inflammasome attenuation correspond to downstream signaling events [12,13].

### 2.2. Concentration-Dependent Effects and the Therapeutic Window of Cyclodextrins

Cyclodextrin bioactivity is concentration- and exposure-dependent, resulting in a functional window where adaptive responses can be observed, whereas excessive lipid extraction may become cytotoxic. Cyclodextrin safety is strongly influenced by the route of administration and dose; while cyclodextrins are generally well tolerated within established ranges, higher exposures can be harmful, including renal toxicity reported for high-dose settings. Therefore, beneficial immunometabolic effects and cytotoxic membrane damage should be interpreted in a context-dependent manner, considering cyclodextrin type, concentration, and exposure time [14,15].

The biological outcome of CD exposure is highly context-dependent and reflects the interplay between cellular lipid burden, metabolic phenotype, and concentration. In lipid-overloaded cells, such as Niemann–Pick type C fibroblasts or cholesterol-rich macrophages within atherosclerotic plaques, cyclodextrin-mediated cholesterol redistribution restores lysosomal function, reactivates autophagic flux, and promotes cell survival while reducing inflammasome activation. In contrast, in cells with normal membrane lipid composition or under high-dose exposure, excessive lipid extraction disrupts membrane integrity, impairs lysosomal stability, and shifts the balance toward apoptotic or pyroptotic cell death. Thus, cyclodextrins act as homeostatic modulators that promote adaptive responses in pathological lipid accumulation but induce cytotoxicity when lipid depletion exceeds the buffering capacity of the cell. This context dependency reconciles the seemingly divergent protective and pro-death effects reported across different experimental systems [16,17,18,19,20].

#### Cyclodextrins and Apoptosis

Apoptosis is the prototypical form of programmed cell death, characterized by caspase activation, DNA fragmentation, and membrane blebbing without eliciting inflammation. Cell membrane microdomains (lipid rafts) rich in cholesterol often serve as platforms for signaling molecules that regulate apoptosis, including death receptors and survival kinases. By virtue of their cholesterol-complexing ability, cyclodextrins can profoundly influence apoptotic signaling. In many cell types, acute cholesterol extraction by MβCD perturbs lipid rafts and has pro-apoptotic effects, whereas in other contexts, cyclodextrins may indirectly inhibit apoptosis by altering cellular metabolism.

One well-documented mechanism is the disruption of pro-survival signaling pathways. For example, targeting cholesterol with β-cyclodextrin was shown to sensitize cancer cells to apoptosis by attenuating Akt signaling [21]. Faes et al. demonstrated that treating cancer cells with β-CD or its methylated derivative disrupted the association between PI3K and Akt in lipid rafts, leading to reduced Akt phosphorylation and diminished anti-apoptotic signaling [22]. This loss of survival signaling lowered the threshold for apoptotic cell death in cancer cells. In a similar vein, removal of membrane cholesterol by MβCD has been found to down-regulate the expression of Bcl-2 (an anti-apoptotic protein) and up-regulate Bax (a pro-apoptotic factor), thereby shifting the balance in favor of apoptosis [23]. Table 1 summarizes such findings, indicating that MβCD and HPβCD can promote apoptosis via raft disruption and mitochondrial pathway activation in various cancer cell lines.

Cyclodextrins themselves can trigger apoptosis in certain contexts. High concentrations of HPβCD were reported to induce significant apoptosis in human leukemia cells and other tumor cells, associated with cell cycle arrest in G_2/M and activation of caspase cascades [43]. In acute myeloid leukemia (AML) and chronic myeloid leukemia models, HPβCD caused dose-dependent apoptosis alongside cholesterol depletion, and it even improved survival in leukemia-bearing mice, highlighting a potential anti-cancer effect of HPβCD as a single agent [44]. These pro-apoptotic effects are often linked to the disruption of cholesterol-rich membranes of the endoplasmic reticulum and mitochondria, which can trigger ER stress and mitochondrial outer membrane permeabilization. Notably, HPβCD showed cytotoxicity against leukemia cells that were resistant to tyrosine kinase inhibitors, suggesting that cholesterol homeostasis is a viable target in drug-resistant cancer stem-like cells [45]. Modified cyclodextrins that target cancer cells can enhance this effect. For instance, folate-appended HPβCD (FA-HPβCD) selectively taken up by folate-receptor-overexpressing leukemia cells was observed to induce apoptosis and autophagy (discussed below) more potently than unmodified HPβCD [46].

In addition to direct induction of apoptosis, cyclodextrins can augment the efficacy of established chemotherapeutic drugs through apoptotic mechanisms. One example is the combination of MβCD with tamoxifen in melanoma treatment. MβCD potentiated tamoxifen-induced apoptosis in melanoma cells and in mouse tumor models by increasing tamoxifen uptake and reinforcing apoptotic signaling [47]. Co-treatment with MβCD enhanced tamoxifen’s effects on cell cycle arrest and apoptosis, leading to greater tumor suppression in vivo compared to tamoxifen alone, an effect reversible by cholesterol supplementation [48]. This suggests that cyclodextrin-mediated cholesterol removal can improve drug permeability and distribution in tumors and disable survival pathways (such as Akt and ERK) that contribute to drug resistance. Similarly, in breast cancer cells, MβCD has been noted to improve the response to trastuzumab and other therapies by modulating the membrane context of receptors.

**Figure 2:**
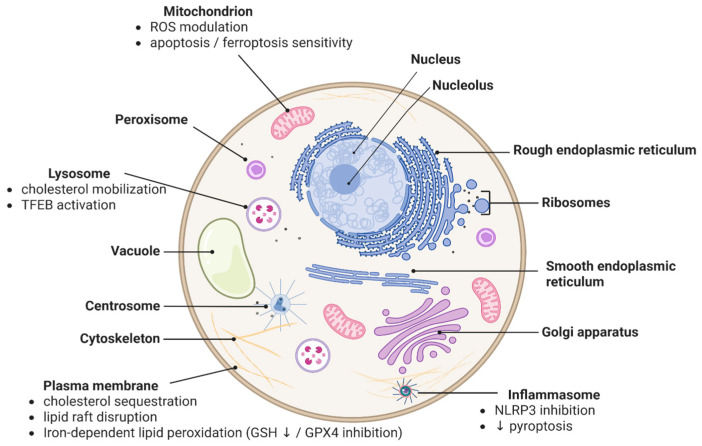
Cellular and organellar sites of cyclodextrin-mediated modulation of regulated cell death pathways. This schematic highlights the key intracellular compartments through which cyclodextrins modulate regulated cell death. Cholesterol sequestration and lipid raft disruption at the plasma membrane promote iron-dependent lipid peroxidation and ferroptosis, while lysosomal cholesterol mobilization and TFEB activation enhance autophagic clearance. Mitochondrial redox modulation influences apoptosis and ferroptosis sensitivity, and inhibition of NLRP3 inflammasome activation attenuates pyroptosis. Created in BioRender. Varut, M. (2026) https://BioRender.com/eoh05u2 (accessed on 7 January 2026).

It is important to note that cyclodextrin-induced apoptosis is not universally beneficial and can affect normal cells under certain conditions. For example, in hepatocytes or other non-malignant cells, excessive cholesterol extraction by HPβCD may trigger apoptosis accompanied by oxidative stress and calcium imbalance. High-dose HPβCD was shown to impede autophagic flux (discussed in the next section) and thereby activate caspase-8, linking autophagy dysfunction to apoptotic cell death in liver cells [49]. These findings highlight a dose-dependent duality: while moderate cholesterol removal can relieve pathological lipid accumulation, overzealous extraction can become cytotoxic. Therefore, the pro-apoptotic action of cyclodextrins must be carefully calibrated in therapeutic contexts to target diseased cells while sparing healthy tissue. The broader cellular and organellar mechanisms through which cyclodextrins influence multiple regulated cell death pathways are summarized in Figure 2.

### 2.3. Cyclodextrins and Autophagy-Dependent Cell Death

Autophagy is a cellular degradation pathway wherein cytosolic components and organelles are sequestered in double-membraned autophagosomes and delivered to lysosomes for turnover. Autophagy generally promotes cell survival under stress by recycling nutrients and removing damaged organelles, but dysregulated or excessive autophagy can lead to type II programmed cell death (often termed autophagy-dependent cell death or “autophagic cell death”). Cyclodextrins have complex, context-dependent effects on autophagy. They can restore autophagic flux in diseases with lysosomal lipid accumulation, yet paradoxically high doses may block autophagosome clearance. Understanding these effects is vital, as autophagy plays dual roles in cell fate and immunity [50].

A striking example comes from Niemann–Pick type C (NPC) disease, a lysosomal storage disorder characterized by cholesterol accumulation and impaired autophagy. HPβCD is known to dramatically improve cholesterol clearance in NPC cells and animal models, and research has uncovered a link to autophagy activation. Treatment of NPC1-mutant cells with HPβCD or its larger-ring analogue HPγCD enhances autophagic function: CDs activate TFEB, a master regulator of lysosomal biogenesis and autophagy, promoting the formation and maturation of autophagosomes [51]. Argüello et al. showed that HPγCD treatment of NPC fibroblasts increased lysosome-ER contacts and autophagic flux, facilitating the redistribution of stored cholesterol out of lysosomes. Moreover, HPβCD was found to stimulate AMPK in NPC cells, which in turn induces autophagy, thereby contributing to cholesterol mobilization from dysfunctional lysosomes [52]. These findings suggest that one therapeutic mechanism of cyclodextrins in storage diseases is the reactivation of autophagy, helping cells to dispose of accumulated substrates. In support of this, a β-cyclodextrin-based polymer conjugate was shown to reduce cholesterol accumulation and restore autophagy in NPC model neurons, correlating with improved cell survival [53].

Cyclodextrins also exhibit autophagy-related effects in other settings. In atherosclerosis, cholesterol crystal deposits in macrophages can block lysosomal function and autophagic flux, contributing to cell death and inflammation. HPβCD, by dissolving cholesterol crystals deposits, has been observed to normalize autophagosome clearance in plaque macrophages, thereby preventing the progression to secondary necrosis or inflammasome activation (overlap with pyroptosis, discussed later) [54]. Furthermore, in neurodegenerative contexts like Alzheimer’s disease, HPβCD has been reported to reduce protein aggregation and promote the clearance of toxic metabolites, possibly by enhancing autophagy and lysosomal exocytosis in neurons and glia [55]. Hein et al. found that HPβCD administration in an Alzheimer mouse model led to decreased amyloid burden and improved cognitive function, coincident with changes in brain lipid profiles and activation of microglial autophagy pathways [56]. While the connection to autophagic cell death per se in these models is still being unraveled, it is clear that cyclodextrins can modulate the autophagy-lysosome system in ways that impact cell survival.

On the other hand, the dose of cyclodextrin is a critical determinant of its autophagic effects. At high concentrations, HPβCD may impair autophagy and trigger cell death. A study in HepG2 liver cells showed that 20 mM HPβCD caused accumulation of LC3-II and p62, markers of blocked autophagy flux, leading to the buildup of autophagosomes that failed to fuse with lysosomes [57]. This blockade was associated with activation of caspase-8 and increased apoptosis, as mentioned earlier. Mechanistically, excessive cyclodextrin might extract too much cholesterol from cellular membranes, including lysosomal membranes, potentially destabilizing them or altering the function of lysosomal enzymes. It might also remove cholesterol needed for autophagosome–lysosome fusion or disrupt membrane curvature dynamics. Interestingly, adding back free cholesterol to HPβCD-treated cells could reverse some of the autophagy block, underscoring cholesterol’s role in membrane trafficking [58]. These observations caution that while cyclodextrins can be pro-autophagic in deficient states, they can become anti-autophagic if overdosed, which could inadvertently promote cell death rather than prevent it.

Another intriguing dimension is the direct induction of autophagy-dependent cell death by targeted cyclodextrins in cancer therapy. Researchers have developed cyclodextrin conjugates that not only deliver drugs but also inherently push cancer cells towards autophagic demise. A notable example is folate-appended HPβCD (FA-HPβCD) tested against acute myeloid leukemia cells. Linder et al. reported that FA-HPβCD, by binding folate receptors abundantly expressed on AML cells, was efficiently internalized and triggered robust autophagy leading to cell death [59]. Treated leukemia cells showed increased LC3-positive autophagosomes and eventual cell death that could be suppressed by autophagy inhibitors, indicating an autophagy-mediated cytotoxic mechanism [60]. In this case, the cyclodextrin itself (with the folate targeting ligand) acted as a trigger for lethal autophagy, possibly by causing intracellular cholesterol redistribution and metabolic stress selectively in the cancer cells. Such an approach blurs the line between using cyclodextrin as a carrier and as an active anticancer agent: here, the cyclodextrin’s inherent activity contributes to killing the cell via autophagy, while its targeting moiety confers selectivity.

### 2.4. Cyclodextrins and Pyroptosis

Pyroptosis is an inflammatory form of programmed cell death typically occurring in immune cells like macrophages in response to infection or danger signals. It is driven by caspase-1 (or caspase-11/4/5 in non-canonical pathways) activation within inflammasomes, leading to the maturation of pro-inflammatory cytokines IL-1β and IL-18 and the cleavage of gasdermin D (GSDMD). Cleaved GSDMD forms pores in the plasma membrane, causing cell swelling, lysis, and release of inflammatory contents. Cholesterol and lipid rafts in the plasma membrane have been implicated in the assembly of certain inflammasome platforms and in GSDMD pore formation. Thus, interventions that alter membrane lipid composition may affect pyroptotic cell death. Indeed, cyclodextrins have emerged as powerful inhibitors of pyroptosis in several disease models by virtue of their cholesterol-depleting action in cell membranes and lysosomes [61].

A paradigm-shifting study in 2025 demonstrated that methyl-β-cyclodextrin can suppress pyroptosis in atherosclerotic plaques, thereby reducing inflammation and disease progression [62]. In a mouse model of atherosclerosis (ApoE mice on a high-fat diet), systemic administration of MβCD markedly reduced the size of arterial plaques by over 50% compared to controls, accompanied by a decrease in lesional macrophage content and inflammatory cytokines [63]. Mechanistic analysis revealed that MβCD inhibited the TLR4/NF-κB/NLRP3 inflammasome pathway and the activation of gasdermin D in plaque macrophages [64]. Treated mice showed lower levels of cleaved GSDMD, IL-1β, and IL-18 in their aortas, indicating that MβCD prevented the macrophages from undergoing pyroptotic death. By extracting cholesterol, MβCD likely disrupted the formation of lipid raft signaling platforms needed for TLR4 and NLRP3 inflammasome assembly. It may also reduce the burden of cholesterol crystals deposits, which are known triggers for NLRP3 inflammasome activation in atherosclerosis. As a result, the cascade leading to caspase-1 activation and GSDMD pore formation was blunted, translating to fewer cells dying violently and releasing cytokines in the plaque environment. The net effect was a shift from a highly inflammatory plaque towards a more stable, quiet lesion. These findings position cyclodextrin as a novel anti-atherosclerotic agent working through an immunometabolic mechanism, breaking the cycle of cholesterol-driven inflammation and cell death [65].

Converging evidence comes from related models of metabolic inflammation. Liu et al. investigated hyperhomocysteinemia-accelerated atherosclerosis and found that macrophage lipid rafts are critical for NOX enzyme clustering, ROS production, and subsequent NLRP3 inflammasome activation. Strikingly, disruption of lipid rafts with MβCD prevented homocysteine-induced NLRP3 activation and pyroptotic cell death in macrophages [66]. MβCD reduced both cytosolic and mitochondrial ROS levels by preventing the assembly of NADPH oxidase subunits in rafts, thereby stopping the upstream trigger of inflammasome activation [67]. It also inhibited the recruitment of NLRP3 and caspase-1 to the raft fractions and decreased the number of macrophages that were double-positive for active caspase-1 and TUNEL (a marker of DNA breaks in pyroptotic cells) [68]. In vivo, mice with high homocysteine on a high-fat diet developed more atherosclerosis with pyroptotic macrophages in lesions; co-treatment with MβCD dramatically lowered IL-1β/IL-18 levels and plaque size despite unchanged homocysteine levels, confirming that MβCD’s anti-atherosclerotic effect was due to inflammation suppression, not lipid-lowering alone [69]. These results reinforce the concept that cyclodextrin’s ability to dismantle lipid raft signaling domains has anti-pyroptotic consequences. By doing so, cyclodextrins break the feed-forward loop where cholesterol crystal deposits and modified lipids drive macrophages to pyroptose, which in turn releases more inflammatory mediators that worsen tissue damage. Beyond metabolic disease, cyclodextrins may have utility in controlling pyroptosis during infections and sepsis. Macrophage pyroptosis is a double-edged sword in infection: it helps clear intracellular pathogens but can also cause septic shock if excessive. There is evidence that certain modified cyclodextrins can bind and neutralize bacterial lipopolysaccharide (LPS, a potent trigger of macrophage TLR4 and pyroptosis) or disrupt host membrane interactions with LPS. For instance, DMβCD was reported to inhibit LPS-induced macrophage activation and protect mice from endotoxic shock, implying a role in preventing pyroptotic or pyroptosis-associated cytokine release [70]. The presumed mechanism is that DMβCD either sequesters LPS away from TLR4 or alters the TLR4 receptor microenvironment, thus dampening the initial inflammatory signal. Additionally, by maintaining plasma membrane integrity (through controlled cholesterol modulation), cyclodextrins might reduce the propensity of GSDMD pores to cause full-blown lysis, instead allowing cells to release IL-1β in a more restrained fashion or undergo apoptosis rather than pyroptosis [71].

It is worth noting that not all effects of cyclodextrins on inflammation are anti-pyroptotic. A study on the effects of HPβCD in a fatty liver inflammation model found an increase in TNF-α and other cytokines with cyclodextrin treatment, suggesting a potential pro-inflammatory effect under certain metabolic conditions [72]. In that study, short-term HPβCD treatment in mice with combined NPC1 haploinsufficiency and high cholesterol burden led to hepatic inflammation despite lowering cholesterol, and in vitro HPβCD exposure could prime macrophages (especially when challenged with secondary stimuli) to produce more TNF-α [73]. This indicates that cyclodextrins might trigger transient inflammasome-independent inflammatory signaling (for example via transient lysosomal stress or membrane perturbation activating NF-κB), even as they block the canonical pyroptosis execution via GSDMD. The timing and context of cyclodextrin exposure likely determine whether the net effect is suppressing or promoting inflammation. In atherosclerotic models, chronic MβCD treatment yields an anti-inflammatory outcome by preventing pyroptosis, whereas an acute high dose in naïve macrophages might cause an inflammatory response (perhaps through assembly of a different inflammasome or release of damage signals due to sudden cholesterol efflux) [74]. Therefore, further research is needed to refine cyclodextrin dosing regimens that maximize pyroptosis inhibition while minimizing any initial pro-inflammatory perturbations.

### 2.5. Cyclodextrins and Ferroptosis

Ferroptosis is a distinct form of regulated cell death characterized by iron-dependent lipid peroxidation of cellular membranes. Unlike apoptosis or pyroptosis, ferroptosis does not involve caspases or membrane pore formation; instead, it results from catastrophic oxidative damage to phospholipids, especially those containing polyunsaturated fatty acids, when cellular antioxidant defenses (like glutathione peroxidase 4, GPX4) are inadequate. The accumulation of lipid peroxides causes loss of membrane integrity and cell death. Because ferroptosis is driven by metabolic factors (iron, reactive oxygen species, and fatty acid composition), there is significant interest in using novel drug delivery systems to modulate this process in diseases such as cancer (where inducing ferroptosis in tumor cells can be beneficial) and neurodegeneration or radiation injury (where inhibiting ferroptosis might be protective). Cyclodextrins have emerged in this arena primarily as enabling delivery platforms for ferroptosis-modulating agents, and secondarily as modulators of lipid composition that might influence susceptibility to ferroptosis [75].

One major application is in cancer therapy: to intentionally trigger ferroptosis in tumor cells. Many ferroptosis inducers (certain chemotherapeutics, natural compounds like artemisinins, or inhibitors of glutathione/GPX4) are hydrophobic and have poor bioavailability. Cyclodextrin-based nanocarriers have been designed to deliver these agents more effectively to tumors. For example, acetalated β-cyclodextrin (Ac-β-CD) nanoparticles were developed to co-deliver dihydroartemisinin (DHA), an iron-activated pro-oxidant, and an iron-containing polymer network to tumors [76]. In this system, the CD-based nanoparticle remains stable in circulation but degrades in the acidic tumor microenvironment, releasing DHA and iron ions specifically in the tumor. The released Fe^2+^/Fe^3+^ catalyzes Fenton reactions that produce hydroxyl radicals, while DHA decomposition yields carbon-centered radicals, together overwhelming the cancer cell’s antioxidant capacity and inducing ferroptosis [77]. Yang et al. demonstrated that these CD-derived nanoparticles potently generated reactive oxygen species (ROS) in tumors and induced ferroptotic death, achieving synergy with conventional chemotherapy. This underscores how chemically modified cyclodextrins (Ac-β-CD in this case) serve as a smart delivery vehicle that unleashes ferroptotic signals in a controlled fashion inside cancer cells. The advantage of using a cyclodextrin scaffold is its biocompatibility and the ease of functionalization (e.g., installing pH-sensitive linkers), which together allow precise spatiotemporal drug release and reduce systemic toxicity [78].

Cyclodextrin inclusion complexes have also been used to solubilize plant-derived or synthetic compounds that can trigger ferroptosis in cancer cells. A recent example is the hydroxypropyl-β-cyclodextrin/thymoquinone (TQ) inclusion complex applied in non-small cell lung cancer (NSCLC) models [79]. Thymoquinone, a hydrophobic phytochemical, has shown anti-cancer effects including the ability to induce oxidative stress in cancer cells. By forming an inclusion complex with HPβCD, the water solubility and bioavailability of TQ were significantly enhanced. Eid et al. found that HPβCD–TQ exhibited stronger anticancer activity than free TQ, and mechanistic studies indicated that it triggered ferroptosis mediated by NF-κB signaling in NSCLC cells [80]. Treated cancer cells showed hallmarks of ferroptosis such as lipid ROS accumulation and cell death that could be rescued by ferroptosis inhibitors, confirming the mode of action. The involvement of NF-κB suggests that TQ/HPβCD might downregulate certain survival pathways or antioxidant responses (NF-κB can regulate genes like SLC7A11 in the cystine/glutamate antiporter system X_c_, important for ferroptosis resistance). By delivering TQ effectively, the cyclodextrin complex was able to tilt the redox balance toward cell death. Such inclusion complexes are relatively simple to prepare (often by co-dissolution or freeze-drying) and underscore the utility of native cyclodextrins in ferroptosis-based therapies.

Beyond delivering inducers of ferroptosis, cyclodextrin formulations have also been employed to deliver ferroptosis inhibitors or mitigate ferroptosis in injury models. For instance, in the context of mitigating radiation damage (where ferroptosis in normal tissues can be a problem), anti-ferroptotic drugs like liproxstatin-1 or ferrostatin-1 have been dissolved or complexed with cyclodextrins to improve their administration. In one study, an anti-ferroptosis drug given to irradiated mice was formulated in 30% HPβCD solution to enhance solubility and stability, which successfully reduced tissue damage post-radiation by blocking ferroptosis in irradiated cells [81]. Similarly, delivering iron chelators to specific organs has benefitted from cyclodextrin-based carriers: e.g., intranasal administration of deferoxamine encapsulated in chitosan/β-cyclodextrin microparticles has been explored to treat neurodegenerative disease by reducing iron-induced oxidative stress in the brain [82]. Cyclodextrin’s ability to traverse certain biological barriers (HPβCD has some blood–brain barrier penetration and when used intranasally can reach the CNS) makes it a candidate to carry ferroptosis-modulating drugs to sites of pathology such as the brain.

It is also noteworthy that cyclodextrin itself can influence cellular lipid composition and thereby possibly the propensity for ferroptosis. A recent fundamental finding by De Gaetano et al. showed that cellular cholesterol can suppress ferroptosis, with higher cholesterol levels protecting against lipid peroxidation, likely by affecting membrane order and the availability of oxidizable fatty acids [83]. In those mechanistic studies, sterols were manipulated in cells using MβCD complexed with cholesterol to either deplete or load cholesterol. Although not the focus of that study’s therapeutic angle, it suggests that using cyclodextrins to modulate membrane cholesterol could indirectly alter ferroptosis sensitivity. Depleting cholesterol (which cyclodextrins do) might actually promote ferroptosis by increasing membrane fluidity and exposure of polyunsaturated lipids to oxidation, or by altering lipid raft-dependent signaling that upregulates antioxidant defenses. Conversely, in pathological states where ferroptosis contributes to damage (like ischemia-reperfusion injury or certain neurodegeneration), one might consider if cyclodextrin-mediated delivery of cholesterol or lipophilic antioxidants to cell membranes could reduce susceptibility. This is a speculative angle, but it points to the deep interplay between lipid biophysics and ferroptotic death [84]. Although direct evidence for CD-induced ferroptosis remains limited, cholesterol depletion and membrane lipid remodeling may indirectly influence ferroptotic susceptibility by altering lipid raft organization and cellular lipid composition. In addition, CD-mediated changes in lysosomal function and redox balance can affect iron handling and lipid peroxidation sensitivity, thereby linking lipid trafficking to ferroptosis regulation in a context-dependent manner. Most reported ferroptotic effects in cyclodextrin-based systems are associated with drug-loaded formulations, supporting a predominantly indirect and context-dependent role of cyclodextrins.

### 2.6. Cyclodextrins and Necroptosis

Necroptosis is a form of regulated necrotic cell death executed by the kinase cascade of RIPK1, RIPK3, and the pseudokinase MLKL. When caspase-8 is inhibited or overwhelmed (preventing apoptosis), death signals like TNF can switch to trigger necroptosis: RIPK3 phosphorylates MLKL, which then oligomerizes and disrupts the plasma membrane, causing a lytic cell death with features similar to unregulated necrosis. Necroptosis is highly pro-inflammatory due to release of damage-associated molecular patterns (DAMPs) and is implicated in various diseases (ischemic injury, neurodegeneration, and certain inflammatory conditions). While cyclodextrins have not been as widely studied in the context of necroptosis as for other RCD forms, emerging evidence suggests they may indirectly modulate necroptotic pathways, particularly through interactions with cellular cholesterol and inflammatory signaling.

One area of overlap is in lysosomal storage disorders (LSDs) such as Niemann–Pick disease type C (NPC), where necroptosis has been identified as a contributor to pathology. NPC involves accumulation of unesterified cholesterol in lysosomes due to NPC1/NPC2 gene defects, leading to neurodegeneration and inflammation. Studies have found that in NPC models, there is evidence of necroptotic cell death in the brain, likely exacerbating neuroinflammation and neuronal loss [85]. Pharmacological inhibition of RIPK1 kinase (a key initiator of necroptosis) showed some benefit in NPC mice, but only a modest extension of lifespan. Notably, when researchers combined a necroptosis inhibitor (RIPK1 inhibitor) with 2-hydroxypropyl-β-cyclodextrin therapy in NPC mice, they observed an additive improvement in survival and disease metrics [86]. HPβCD on its own is known to markedly slow NPC disease progression by clearing lysosomal cholesterol; however, it does not directly target cell death pathways. The addition of a RIPK1 inhibitor further reduced neuroinflammation and cell death, suggesting that necroptosis was indeed occurring in NPC and that HPβCD and necroptosis inhibition work via complementary mechanisms [87]. Interestingly, genetic knockout of RIPK3 (downstream in necroptosis) did not confer the same survival benefit in NPC mice as RIPK1 blockade, hinting that RIPK1 might be contributing to NPC pathology partly through necroptosis-independent inflammation (RIPK1 has scaffolding roles in inflammatory signaling apart from executing necroptosis). Nonetheless, these findings imply that HPβCD’s therapeutic effects in NPC could be augmented by concurrently dampening necroptosis, a strategy that may hold for other LSDs where inflammation and cell death intersect.

Cyclodextrins might also influence necroptosis by modulating upstream signals such as TNF receptor signaling complexes or the bioavailability of death ligands. TNF-induced necroptosis often depends on membrane microdomains for the assembly of the necrosome complex after caspase-8 inhibition. Cholesterol-rich domains may facilitate the clustering of TNF receptor 1 or other receptors like Toll-like receptors that, upon zVAD (pan-caspase inhibitor) treatment or viral infection, can lead to necroptosis. By altering membrane organization, cyclodextrins could in theory disrupt these platforms. For example, removal of cholesterol by MβCD has been shown to inhibit TNF-induced signaling to certain pathways (like NF-κB and MAPKs); if the necroptosis-inducing complex also requires such membrane localization, MβCD might reduce necroptosis initiation. However, direct evidence of MβCD preventing necroptosis is sparse compared to its clear effects on pyroptosis or apoptosis. Some hints come from cell culture studies where MβCD reduced the formation of amyloid oligomer–lipid complexes that can cause neuronal necroptosis, or where it preserved cell viability in contexts of toxin-induced necrotic death by stabilizing membranes.

Another angle is the potential of cyclodextrins to mitigate downstream consequences of necroptosis. Necroptotic cells release danger signals like HMGB1, nucleic acids, and cytokines that amplify tissue injury. Cyclodextrins have been shown in other contexts to bind or neutralize certain hydrophobic DAMPs. For instance, CDs can bind oxidized lipids that act as DAMPs, or LPS as mentioned. In the aftermath of necroptosis (which often involves membrane rupture similar to pyroptosis), cyclodextrins might soak up some of the released hydrophobic mediators, thereby dampening the secondary inflammatory response. Though not a direct block of necroptosis, this could improve outcomes. In models of sterile inflammation like ischemia-reperfusion injury (where necroptosis plays a role in cell death after blood flow returns), cyclodextrin treatment has been observed to reduce tissue damage and fibrosis, possibly by clearing toxic lipid metabolites. Moerke et al. found that HPβCD protected kidney cells (podocytes) from diabetic injury by reducing lipid buildup and inflammasome activation; considering necroptosis is also implicated in diabetic nephropathy, one wonders if HPβCD indirectly reduced necroptotic signaling in podocytes by keeping their cholesterol and ceramide levels low [88]. Direct experimental evidence for cyclodextrin-mediated necroptosis regulation remains limited. Necroptosis is tightly connected to membrane signaling platforms and inflammatory receptor activation, both of which depend on cholesterol-rich microdomains. By disrupting lipid raft integrity, cyclodextrins may indirectly influence death receptor clustering and downstream RIPK1/RIPK3 signaling. Furthermore, their ability to attenuate inflammasome activation and restore lysosomal function suggests an indirect regulatory role in necroptosis-associated inflammatory cell death. These observations support a predominantly indirect, membrane-organization-dependent effect rather than direct modulation of the necroptotic machinery.

## 3. Immunometabolism and Macrophage Polarization Modulated by Cyclodextrins

The term immunometabolism refers to the interplay between metabolic processes and immune cell function. Macrophages, as central players in innate immunity and inflammation, are a prime example: they adopt distinct polarization states (simplistically categorized as pro-inflammatory M1 vs. anti-inflammatory M2) with characteristic metabolic profiles. M1 macrophages rely on glycolysis and produce high levels of inflammatory mediators (IL-1β, TNFα, ROS), often contributing to tissue damage. M2 macrophages favor oxidative metabolism and participate in tissue repair and resolution of inflammation. Cholesterol metabolism is a crucial factor in macrophage biology, as these cells ingest lipids (becoming foam cells in atherosclerosis) and the accumulation of cholesterol or oxysterols can trigger inflammatory pathways like inflammasomes. Cyclodextrins, by virtue of modulating cholesterol and lipid handling in cells, have a profound impact on macrophage immunometabolism and polarization state.

A hallmark study by Chen et al. showed that cyclodextrin promotes atherosclerosis regression via macrophage reprogramming [89]. In that work, a 2-hydroxypropyl-β-cyclodextrin treatment not only reduced arterial plaque size but also shifted macrophages within the plaque from a pro-inflammatory state toward a reparative state. Specifically, HPβCD was found to stimulate cholesterol efflux pathways in macrophages (upregulating ATP-binding cassette transporters like ABCA1 and ABCG1 via activation of liver X receptors, LXRs), thereby unloading excess cholesterol from foam cells. This process inherently drives macrophages toward an M2-like phenotype, since cholesterol-laden macrophages tend to be inflammatory (cholesterol crystals deposits activate NLRP3 inflammasomes driving IL-1β release, a hallmark of M1 polarization). By clearing cholesterol, cyclodextrin-treated macrophages showed reduced inflammasome activation and lower expression of M1 markers, and instead exhibited markers associated with alternative (M2) activation such as increased arginase-1 and IL-10 in some reports [90]. The reprogramming of macrophages was integral to plaque regression: macrophages with normalized cholesterol content can enact efferocytosis (clearance of dead cells) and produce resolving factors, leading to shrinkage and stabilization of plaques. Thus, cyclodextrin acted as an immunometabolic modulator, turning destructive macrophages into healing ones.

The immunometabolic reprogramming described above is supported by functional metabolic assays that directly quantify macrophage bioenergetics. In experimental systems, Seahorse extracellular flux analysis has been used to measure the oxygen consumption rate and extracellular acidification rate, allowing discrimination between oxidative-phosphorylation- and glycolysis-dependent states. In addition, assessment of mitochondrial membrane potential, ATP production, fatty acid oxidation, and cholesterol efflux provides mechanistic evidence for the metabolic shift associated with macrophage polarization. Integration of these bioenergetic readouts with phenotypic markers strengthens the interpretation that CD-induced cholesterol redistribution drives a transition toward a less inflammatory, pro-resolving macrophage state.

The role of cyclodextrins in macrophage polarization extends beyond atherosclerosis. In the context of sepsis, where a hyper-inflammatory macrophage response can be fatal, cyclodextrin-based delivery of microRNAs has been employed to alter macrophage polarization. Ding et al. developed a β-CD-based pH-responsive nanoparticle to deliver miR-223 specifically to macrophages in sepsis [91]. MiR-223 is a microRNA known to suppress pro-inflammatory gene expression and promote the switch of macrophages from M1 to M2 phenotype. The cyclodextrin-polymer nanoparticle (β-CD–PDPA/DSPE-PEG) was designed to target acidosis in inflamed tissues, releasing miR-223 inside macrophages at the infection sites. The result was a significant reduction in systemic inflammation and improved survival in septic mice, attributable to miR-223 driving macrophage polarization toward an anti-inflammatory, M2 state [92]. The treated macrophages showed lower NF-κB activity and decreased TNFα/iNOS (M1 markers), while increasing IL-10 and arginase-1 (M2 markers) via the miR-223 targeting of Pknox1 and downstream effects [93]. This innovative approach highlights how cyclodextrins can serve as vehicles to reprogram immune cell metabolism and function in acute inflammatory diseases.

Cyclodextrin nanoparticles themselves (even without a miRNA cargo) have been noted to exert anti-inflammatory effects on macrophages. For instance, certain β-CD-based nanosponges and nanoparticles have been shown to reduce macrophage secretion of cytokines in rheumatoid arthritis models, correlating with a shift to an M2-like gene profile [94]. These effects may stem from the nanoparticle scavenging inflammatory lipids in the synovial environment or delivering soothing biomolecules. In one study, a cyclodextrin nanoparticle loaded with an anti-miR (against miR-33, a microRNA that suppresses cholesterol efflux) was used to treat atherosclerotic mice: the anti–miR-33/CD nanoparticle therapy upregulated cholesterol efflux in lesional macrophages and modulated them towards an M2 phenotype, enhancing plaque stability. MiR-33 normally keeps macrophages in a lipid-laden, inflammatory state by inhibiting ABCA1; blocking it via a CD-based nanotherapy freed the macrophages to offload cholesterol and adopt a reparative program [95]. This not only validates the immunometabolic mechanism (more cholesterol efflux leading toless inflammation) but also demonstrates the precision with which cyclodextrin carriers can target molecular switches of macrophage polarization.

Additionally, cyclodextrins can influence the recruitment and clearance of macrophages. In chronic inflammatory diseases, continuous recruitment of monocytes that differentiate into M1 macrophages sustains pathology. Cyclodextrin treatments that reduce local inflammation (by preventing pyroptosis or releasing pro-resolving factors from M2 macrophages) can create a positive feedback loop: fewer inflammatory signals mean less monocyte chemoattractant protein-1 (MCP-1) and other chemokines, thereby reducing infiltration of fresh M1 cells. Some evidence of this was seen in the hyperhomocysteinemia study: MβCD treatment led to reduced macrophage recruitment into lesions, likely because the inflammation (and hence chemokine expression) was curtailed [96]. Another example is in NASH (nonalcoholic steatohepatitis) models, where cyclodextrin has been shown to decrease Kupffer cell activation and shift the liver macrophage pool to an alternative activation state, improving liver insulin sensitivity and fibrosis outcomes [97].

On the flip side, as mentioned earlier, an acute exposure of macrophages to cyclodextrins in certain contexts might provoke a transient M1-like response. Petrova et al. observed that bone marrow-derived macrophages briefly exposed to HPβCD upregulated TNFα and IL-1β production upon LPS stimulation, suggesting a priming effect rather than a tolerant effect [98]. This could be due to cyclodextrin causing lysosomal cholesterol exit, activating TFEB and possibly inducing cytokine gene programs (since TFEB has connections to inflammation as well). However, this pro-inflammatory priming seems context-dependent and may be overshadowed by the longer-term benefits of reducing cholesterol. The net impact in vivo tends to favor anti-inflammatory outcomes of cyclodextrin therapy, as evidenced by improved histology in disease models.

From a pharmaceutical perspective, immunometabolic reprogramming by cyclodextrins underscores the importance of formulation-dependent biodistribution, macrophage targeting, and release kinetics. Differences between free cyclodextrins, inclusion complexes, and nanoparticulate systems critically influence cellular uptake pathways, intracellular cholesterol handling, and downstream regulated cell death responses. Rational design of cyclodextrin-based systems must therefore consider not only drug solubilization, but also cell-type-specific metabolic states, immune cell polarization, and context-dependent activation or suppression of regulated cell death pathways.

To clearly distinguish intrinsic cyclodextrin bioactivity from carrier-mediated effects, it is important to consider whether the observed biological response occurs in the absence or presence of a pharmacological cargo. Native or unloaded cyclodextrins primarily act through cholesterol redistribution, lysosomal functional changes, and membrane remodeling, thereby directly modulating regulated cell death and immunometabolic pathways. In contrast, cyclodextrin-based inclusion complexes, polymers, and nanoparticles mainly exert their effects by improving the solubility, stability, cellular uptake, or intracellular delivery of active compounds. In these systems, the modulation of cell death is predominantly drug-driven, while the cyclodextrin scaffold provides pharmacokinetic and targeting advantages. Therefore, the biological outcome reflects the combined contribution of cyclodextrin intrinsic activity and formulation-dependent drug action.

## 4. Applications of Cyclodextrin-Modulated Cell Death in Disease

**Figure 3:**
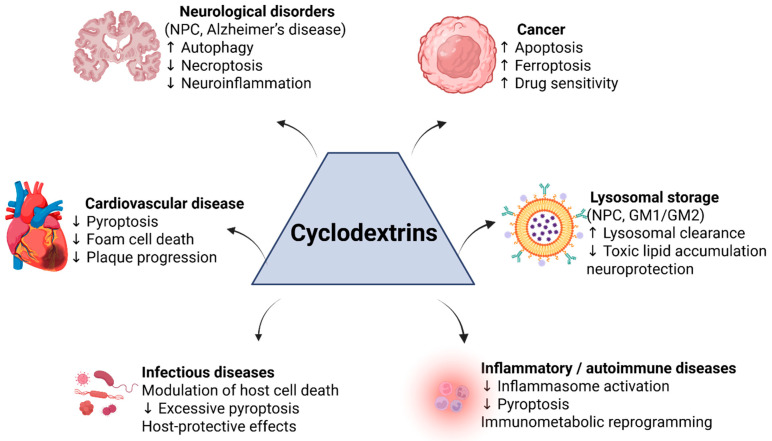
Schematic representation of the molecular pathways affected by cyclodextrin-mediated cholesterol depletion, including lysosomal function, autophagy, macrophage metabolism, and inflammasome signaling. Created in BioRender. Varut, M. (2026) https://BioRender.com/d1d7bjw (accessed on 5 January 2026).

CDs exert therapeutic effects either through their intrinsic cholesterol-modulating bioactivity or as components of drug delivery systems that enhance the efficacy of cell-death-targeting agents. In this section, we explore how cyclodextrin-based strategies have been applied or proposed in major disease categories—cancer, cardiovascular disease, neurological disorders, inflammatory and autoimmune diseases, infectious diseases, and lysosomal storage disorders—highlighting the role of apoptosis, autophagy, pyroptosis, ferroptosis, or necroptosis modulation in each context. CD-mediated cholesterol depletion promotes lysosomal cholesterol mobilization and activates TFEB-dependent autophagy. These events drive macrophage metabolic reprogramming and attenuate inflammasome activation, thereby influencing regulated cell death pathways in cholesterol-driven pathologies such as atherosclerosis and lysosomal storage disorders (Figure 3).

### 4.1. Cancer

In oncology, the primary goals are to induce death of cancer cells (preferably immunogenic forms of death that stimulate anti-tumor immunity) and to overcome resistance to therapy.

HPβCD can directly trigger apoptosis in cancer cells by disrupting cholesterol-dependent survival pathways. This property has been leveraged in hematological malignancies: for example, HPβCD showed potent anti-leukemic activity, inducing apoptosis in AML and CML cells and prolonging survival in mouse leukemia models [99]. Unlike many conventional chemotherapies that cancer cells often develop resistance to, cyclodextrin’s mechanism—targeting cholesterol metabolism—is orthogonal and can kill even cells with mutations (T315I BCR-ABL) that make them drug-resistant [100]. Similarly, FA-HPβCD induced autophagic cell death in AML as a targeted anti-cancer approach [101]. In solid tumors, while systemic HPβCD alone is not yet used clinically as an anti-cancer drug, local effects are noted: e.g., intratumoral injection of cyclodextrin in some experimental models reduced tumor growth, possibly by causing cancer cell apoptosis and altering the tumor microenvironment (TME) cholesterol content.

Cancer cells often evade apoptosis via upregulated survival pathways or drug efflux pumps. Cyclodextrins can counteract some of these evasion tactics. MβCD’s ability to increase drug penetration into cells and tumors has been shown with tamoxifen in melanoma (augmenting tamoxifen-induced apoptosis) [102] and with other agents like doxorubicin or EGFR inhibitors in various tumor models. By depleting membrane cholesterol, cyclodextrins can downregulate drug efflux transporters embedded in lipid rafts and improve the intracellular retention of chemotherapeutics. There is also evidence that combining cyclodextrins with photodynamic therapy or radiotherapy can enhance cancer cell killing, as cholesterol depletion may reduce the threshold for oxidative damage (potentially tipping cells into ferroptosis or apoptosis more easily). Some nanomedicines incorporate CDs to this end: a β-CD polymer conjugated with camptothecin (CRLX101) was designed to slowly release the drug inside cancer cells, overcoming solubility issues and achieving sustained DNA damage in tumors. CRLX101 reached Phase 2 trials for multiple cancers, demonstrating that cyclodextrin-based carriers can be translated into clinical oncology. While CRLX101’s mechanism is primarily through apoptosis induction via camptothecin (a topoisomerase inhibitor), the cyclodextrin polymer itself aided in preferential tumor accumulation and prolonged drug exposure, indirectly influencing the stress responses (possibly even ferroptosis in hypoxic cores as some studies suggest camptothecin may induce lipid peroxidation) [103].

Nanoparticle-mediated delivery of miR-223 has emerged as a promising strategy for macrophage reprogramming and immune modulation. A cyclodextrin-based pH-responsive nanoplatform enabling intracellular release of miR-223 and functional polarization of macrophages was recently reported by Ding et al., demonstrating efficient cytosolic delivery and therapeutic efficacy in vivo. Although this system was primarily evaluated in inflammatory settings, it highlights the potential of cyclodextrin carriers to modulate macrophage phenotype through microRNA delivery. In parallel, other non-cyclodextrin nanocarriers have confirmed that miR-223 nanotherapy can reshape immune responses and regulate macrophage activation in both inflammatory and tumor microenvironments, supporting the broader concept of microRNA-based immunometabolic reprogramming [91,104]. On the other hand, CDs can be used to induce immunogenic cell death forms like pyroptosis or ferroptosis in cancer cells, which release damage signals and neoantigens that stimulate dendritic cells and T cells. The DHA-loaded CD nanomedicine that causes ferroptosis in tumors is a case in point: the resulting tumor cell death can be inflammatory and recruit immune cells to clean up the tumor, potentially synergizing with checkpoint inhibitors. There is burgeoning interest in using cyclodextrin nanoparticles to deliver STING agonists or other immune modulators into the TME, capitalizing on their ability to target phagocytic cells.

In clinical development, cyclodextrin-containing therapies for cancer include: CRLX101 (now known as AZD0466 or other code names in newer formulations) for solid tumors [105] and cyclodextrin-based siRNA delivery systems (the first in-human siRNA nanoparticle, CALAA-01, was a cyclodextrin polymer targeting tumor transferrin receptors—while not explicitly modulating cell death, it validated the platform) [106]. Cyclodextrin-based systems have also been explored for the delivery of classical chemotherapeutics such as paclitaxel using β-cyclodextrin polymeric nanoparticles, which improve drug solubility and tumor accumulation [107]. In contrast, sulfobutylether-β-cyclodextrin (Captisol) is primarily used as a solubilizing excipient in clinically approved intravenous formulations, such as carfilzomib, rather than as a nanocarrier [108]. Moreover, HPβCD is being evaluated as an adjuvant in certain chemotherapy regimens to see if lowering tumor cholesterol makes conventional drugs more effective (there are preclinical hints of this in breast cancer). Cyclodextrin-based nanomedicines such as CRLX101, together with other oncological examples, highlight the successful transition of these systems from theoretical design to clinical investigation. [109].

### 4.2. Cardiovascular Diseases

Cyclodextrins have shown promise in multiple cardiovascular contexts, primarily through their ability to reduce pathological lipid accumulation and associated cell death/inflammation:

- Atherosclerosis: This is the marquee application, where cyclodextrins have been demonstrated to regress plaques in animal models. HPβCD and MβCD reduce cholesterol in arterial walls by mobilizing it for efflux via HDL pathways [110]. More importantly, as discussed in the pyroptosis section, they reduce macrophage pyroptosis and necrotic core formation within plaques by suppressing inflammasome activation and GSDMD pore formation [111]. The macrophage reprogramming effect also leads to more efficient clearance of dead cells (efferocytosis) and less plaque instability. Impressively, in mice, HPβCD given weekly led to smaller aortic root plaques and was associated with a more fibrous, stable plaque phenotype with fewer inflammatory cells [112]. This kind of plaque remodeling is the goal of advanced atherosclerosis therapy. Encouraged by these findings, there is interest in moving HPβCD or analogues into clinical trials for atherosclerosis or coronary artery disease. One biotech effort (mentioned in press as “Cholesterol efflux mediators”) involves engineered cyclodextrin dimers (such as a dimer known as DRME-β-CD) that have enhanced ability to bind 7-ketocholesterol and other oxidized sterols in plaques [113]. These specialized CDs aim to selectively target arterial plaques and are in preclinical development.
- Myocardial Infarction and Heart Failure: Following a heart attack, damage-associated molecular patterns and cell death (including pyroptosis and necroptosis) contribute to adverse remodeling and heart failure [114]. Cyclodextrin-mediated mobilization of cholesterol and suppression of inflammatory signaling has been shown to promote cardiovascular tissue remodeling and macrophage reprogramming in vivo, suggesting a potential cardioprotective role in ischemic injury [109]. HPβCD has been shown to suppress cholesterol-crystal-driven inflammasome activation and IL-1β production in atherosclerotic lesions. Given the recognized role of inflammasome signaling in post-infarction cardiac inflammation, similar mechanisms may contribute to cardioprotection, although this has not yet been directly demonstrated in myocardial infarction models [115]. Cyclodextrins may also help prevent microvascular dysfunction by clearing cholesterol from endothelial cells [116].
- Abdominal Aortic Aneurysm (AAA): AAA involves chronic aortic inflammation, extracellular matrix degradation, and vascular smooth muscle cell death (sometimes via autophagy and apoptosis). Lu et al. study reported that HPβCD activated TFEB and autophagy in vascular cells, which was protective against aneurysm formation [117]. By enhancing autophagic clearance of degenerated organelles and possibly reducing apoptosis of smooth muscle cells, HPβCD-treated mice had a lower incidence of aneurysm. This suggests cyclodextrins might strengthen the vessel wall by promoting cell survival and anti-inflammatory macrophage phenotypes (since macrophages in AAA contribute to wall degeneration). AAA is an example where cyclodextrin’s activation of autophagy (beneficial) and reduction of inflammasome activity can converge to a therapeutic effect.
- Diabetic Cardiomyopathy and Nephropathy: In diabetic models, as mentioned, HPβCD protected kidney podocytes by reducing lipid-driven NLRP3 activation and consequent cell injury, which is relevant to diabetic kidney disease. Similarly, diabetic heart tissue accumulates lipid droplets and can undergo ferroptosis due to high oxidative stress. Cyclodextrins might alleviate lipotoxicity in such settings. Indeed, a company (ZyVersa Therapeutics) is looking at a cyclodextrin (VAR 200) for focal segmental glomerulosclerosis (a kidney disease associated with lipid-laden podocytes), which is conceptually similar to addressing diabetic nephropathy by enhancing cholesterol efflux [118].

In terms of clinical status, intravenous HPβCD (Trappsol Cyclo) is not yet officially indicated for cardiovascular disease, but safety data from NPC patients (who often receive IV or intrathecal HPβCD) provide some basis to proceed. The key will be demonstrating benefit in patients with atherosclerosis. Since statins already manage cholesterol levels, cyclodextrin would be targeting the inflammatory residual risk by clearing cholesterol crystal deposits and dead cell debris in plaques. There is speculation about combining HPβCD with statin therapy to see additive effects on plaque regression. It is noteworthy that in the animal studies, cyclodextrin could even regress existing plaques, an ability not well-established for conventional lipid-lowering therapy alone [119].

### 4.3. Neurological Disorders

Neurological diseases present a challenge for drug delivery due to the blood–brain barrier (BBB), but cyclodextrins have been central to one of the most promising experimental therapies for a neurodegenerative condition.

Niemann–Pick type C (NPC), a fatal pediatric neurodegenerative disease, has been a primary focus for cyclodextrin therapy. HPβCD (VTS-270) demonstrated the ability to delay neurological progression in NPC1 patients when administered intrathecally (directly into the cerebrospinal fluid) in a Phase 1/2 trial [120]. In a randomized, double-blind Phase 1 trial in adults with NPC1, HPβCD treatment was associated with biomarker and tissue evidence of reduced cholesterol storage and with reported stabilization or slower clinical decline in domains such as ambulation, cognition, and swallowing over extended follow-up, warranting confirmation in controlled trials [121]. While not a cure, this was a significant breakthrough given the lack of other effective treatments. HPβCD (both VTS-270 and a similar product Trappsol Cyclo by another company) has since progressed through Phase 2/3 trials in NPC. The mechanism involves HPβCD entering cells (possibly via endocytosis), binding cholesterol in lysosomes, and facilitating its export and excretion. This reduces the downstream toxic cascades—including necroptosis and neuroinflammation as discussed [122]—thereby preserving neuronal function. The clinical use of cyclodextrin in NPC is the most advanced example of repurposing an excipient as a therapeutic for a CNS disorder. Regulatory approval is being sought, and compassionate use programs have been ongoing (with some NPC patients on cyclodextrin therapy for over 5 years, which has also revealed safety considerations like hearing loss at high doses).

Alzheimer’s disease (AD) is characterized by amyloid-beta plaques, tau tangles, and cholesterol-rich lipid dysregulation in the brain. Because apolipoprotein E and cholesterol metabolism are linked to AD risk, there has been interest in HPβCD for AD. Preclinical studies showed that HPβCD given to AD model mice reduced amyloid deposition and improved cognitive performance. The proposed mechanism is multifaceted: HPβCD may enhance the clearance of amyloid by microglia (improving their cholesterol metabolism and phagocytic capacity), reduce cholesterol accumulation in neurons (which can favor processing of amyloid precursor protein down amyloidogenic pathways), and even activate autophagy to clear protein aggregates. Furthermore, some neuroprotective effect might come from anti-inflammatory action, as HPβCD could reduce microglial inflammasome activation akin to what it does in macrophages. On the clinical front, Cyclo Therapeutics has initiated a Phase 2 trial of IV Trappsol Cyclo in early Alzheimer’s disease (NCT05607615), banking on the rationale that improving brain cholesterol turnover could slow cognitive decline. It is a novel approach, and results are eagerly awaited [123].

There are smaller-scale studies or case reports exploring cyclodextrins in conditions like Huntington’s disease, Parkinson’s disease, and amyotrophic lateral sclerosis (ALS), primarily in animal models. In Huntington’s, cyclodextrin treatment reduced huntingtin aggregates and neuroinflammation in mice, again hinting at improved autophagic clearance. In ALS models, there is speculation that CD could help remove aberrant lipid accumulations in spinal cord cells. Another LSD, GM2 gangliosidosis (Tay–Sachs disease), has been discussed as a potential target for cyclodextrin therapy since ganglioside storage might be mitigated by upregulating lysosomal exocytosis and autophagy via TFEB activation. However, those remain exploratory [124].

One important note is that while cyclodextrins do not readily cross the intact BBB when given IV (they are large and hydrophilic), in disease states, the barrier may be somewhat compromised, and direct CNS delivery (intrathecal) bypasses it. Ototoxicity (damage to cochlear hair cells) has been observed in animal models (cats) and some human NPC patients on chronic HPβCD, likely due to cyclodextrin’s cholesterol extracting effect in those delicate cells [125]. This underscores that while cyclodextrin is beneficial in clearing storage material, neurons, and sensory cells still need cholesterol for normal function—a balance that dosing regimens must manage.

### 4.4. Inflammatory and Autoimmune Diseases

Chronic inflammatory diseases, including autoimmune conditions, are characterized by persistent activation of immune cells, often with an imbalance in cell death (e.g., defective clearance of apoptotic cells or excessive inflammasome activity). In rheumatoid arthritis (RA), macrophages and synovial fibroblasts sustain inflammation in the joints. Cyclodextrin nanoparticles have been investigated to deliver anti-inflammatory agents to synovial macrophages. For example, a β-CD nanoparticle carrying a STAT3 decoy or an NF-κB inhibitor could induce apoptosis in M1-like synovial macrophages or reprogram them to an M2 state, reducing joint inflammation. One study created cyclodextrin-based nanogels loaded with methotrexate that specifically targeted inflamed joints and demonstrated improved arthritis outcomes with lower systemic toxicity [126]. Another used CD nanoparticles to deliver microRNA-155 inhibitors to macrophages, curbing the inflammatory phenotype. Additionally, HPβCD itself has a mild cholesterol-lowering effect that could benefit RA patients, as RA is associated with altered lipid profiles and possibly cholesterol crystal formation in joints. [127]

The role of inflammasomes and pyroptosis in multiple sclerosis (MS) pathology (particularly in microglia) has been an area of research. Cyclodextrins might be able to reduce the activation of microglial inflammasomes by removing cholesterol crystal deposits or oxidized lipids from areas of myelin damage. There was a small study where HPβCD was intrathecally administered in a model of demyelination; it resulted in reduced lipid accumulation in microglia and a shift to a less inflammatory state, although translating that to human MS is far off [128]. These are auto-inflammatory diseases driven by excessive inflammasome activation (like CAPS—cryopyrin-associated periodic syndromes). While specific inflammasome inhibitors are in use, like IL-1 blockers, one could envision using cyclodextrins to reduce triggers (if they are cholesterol or urate crystals in gout). In fact, in gout where monosodium urate crystals deposits cause NLRP3-driven pyroptosis in joints, a local injection of HPβCD might dissolve some crystals or mitigate the inflammation. This remains hypothetical but is an intriguing possibility given cyclodextrin’s success with cholesterol crystals deposits [129].

Gut inflammation and cell death (particularly necroptosis of intestinal epithelial cells and pyroptosis of infiltrating macrophages) contribute to IBD pathology. There is preliminary exploration of oral cyclodextrin formulations to deliver anti-inflammatory compounds or to bind bile acids that serve as inflammatory stimuli. For example, β-cyclodextrin has been tested as a carrier for butyrate (an anti-inflammatory short-chain fatty acid) to the colon, enhancing its local effects. Also, a recent approach used a β-CD derivative to scavenge excess reactive oxygen species in colitis, indirectly protecting gut epithelial cells from ferroptosis [130].

While none of these are yet in advanced clinical trials for autoimmune diseases, the versatility of cyclodextrin carriers is being harnessed to fine-tune immune cell fate. The anti-miR33 and miR-223 examples for atherosclerosis and sepsis, respectively, could be paralleled in autoimmune settings by delivering, say, a microRNA or siRNA that corrects an immune imbalance (like silencing a pro-inflammatory cytokine or a cell death mediator specifically in macrophages or dendritic cells). Table 2 includes RA and other inflammatory conditions with the relevant cyclodextrin approach used.

### 4.5. Infectious Diseases

Infections can trigger extensive cell death (e.g., pyroptosis during septicemia, T-cell apoptosis during HIV infection), and pathogens often exploit or are protected by host cell lipids. Cyclodextrins have some niche but significant roles:

- Sepsis and Septic Shock: Sepsis is an overwhelming inflammatory response often due to bacterial endotoxins (LPS) activating macrophages and causing pyroptosis and cytokine storm. As previously noted, modified cyclodextrins like DMβCD can neutralize LPS and attenuate endotoxin shock in mice by preventing macrophage hyperactivation [159]. Also, the miR-223 cyclodextrin nanoparticle that shifts macrophages to anti-inflammatory mode significantly improved survival in a mouse model of polymicrobial sepsis, essentially “reprogramming” the immune response to be less damaging. These are promising adjunctive strategies to antibiotics, aiming to prevent organ damage caused by the host’s own excessive cell death and inflammation. Clinically, sepsis management could benefit from such immunomodulation; however, safety in critically ill patients would have to be proven. It is notable that CDs also might act as hemoperfusion agents—there was research into cyclodextrin polymers embedded in filters to cleanse blood of endotoxins or cytokines during dialysis for sepsis [160].
- Viral Infections: Many enveloped viruses, including HIV and hepatitis C virus, depend on cholesterol in the viral envelope and host cell membrane for entry and budding. Methyl-β-cyclodextrin is widely used in vitro to extract membrane cholesterol, which renders virions non-infectious by preventing membrane fusion and virus–cell binding [161]. Some cyclodextrin derivatives (2,6-di-*O*-methyl-β-CD, etc.) were reported to reduce the infectivity of SARS-CoV-2 and influenza virus, and they are being looked at as potential broad-spectrum antivirals that act on the host membrane rather than the virus genome (Table 2). Moreover, CDs can be used to deliver antiviral drugs: for example, HPαCD was tested as a nasal spray excipient with an antiviral compound to enhance its distribution on the mucosa. In chronic viral infections like hepatitis B or C, liver damage can result from pyroptosis and apoptosis of hepatocytes driven by immune cells. Cyclodextrins could theoretically mitigate the collateral damage by reducing inflammasome activation in Kupffer cells (liver macrophages) or by protecting hepatocytes from lipid peroxidation (since NASH and hepatitis often intersect, an anti-ferroptotic approach with CDs might be relevant) [162].
- Bacterial Toxins Several bacterial pore-forming toxins, including *Staphylococcus aureus* α-toxin and cholesterol-dependent cytolysins such as streptolysin O, perforate host cell membranes and induce cell lysis. β-Cyclodextrin derivatives have been investigated as channel-blocking inhibitors that interfere with pore formation by binding within the oligomeric toxin pore. These multivalent scaffolds, particularly β-CD-based constructs, can inhibit toxin-mediated membrane permeabilization and thereby protect cells from toxin-induced necrotic damage [163].
- Parasitic and Fungal Infections: Certain antiparasitic and antifungal drugs use cyclodextrin formulations (e.g., HPβCD is used to solubilize the antifungal itraconazole for IV use). While in these cases the CD’s role is as a delivery agent rather than directly affecting cell death pathways, the improved drug delivery can lead to better parasite/fungus killing and possibly reduced host cell toxicity. There is some indication that cyclodextrin complexes with antimalarial drugs (like artemisinin derivatives) could enhance their ability to induce programmed death in the parasite [164].

### 4.6. Lysosomal Storage Disorders (LSDs)

Wolman disease and cholesteryl ester storage disease (CESD) are caused by lysosomal acid lipase deficiency leading to buildup of cholesterol esters and triglycerides in lysosomes. A 2017 review by de las Heras et al. pointed to cyclodextrins as a potential approach to mobilize cholesterol in these diseases, similar to NPC [165] Cyclodextrins, particularly HP-β-CD, are being actively investigated as therapeutic agents for lysosomal storage disorders. In Niemann–Pick disease type C, HP-β-CD enters cells via endocytosis, localizes to the endo-lysosomal compartment, binds unesterified cholesterol, and facilitates its efflux, thereby partially compensating for defective NPC1/NPC2-mediated transport. Preclinical studies and compassionate-use and clinical trials have demonstrated beneficial effects, although issues such as limited blood–brain barrier penetration and ototoxicity remain to be resolved [166].

In the feline NPC1 model, GM2 and GM3 gangliosides accumulated early in neurons and were markedly reduced following intracisternal HPβCD administration, in parallel with decreased cholesterol storage, preservation of Purkinje cells, and improved neurological function. These findings indicate that normalization of lysosomal cholesterol trafficking is accompanied by a secondary correction of ganglioside storage [167,168].

In LSDs, cyclodextrins are one of the few interventions that can directly address the storage pathology at the cellular level, which is why they have generated hope. The “conditional approval” or expanded access of HPβCD for NPC by certain agencies is a milestone in this field. Table 3 lists NPC and related disorders under cyclodextrin clinical development. With luck, success in NPC could pave the way for cyclodextrins becoming part of standard care in other storage diseases, especially those with significant neurological involvement where small molecules have an advantage over large protein enzymes (which often cannot cross the BBB) [169].

The breadth of applications above demonstrates cyclodextrins’ transition from lab curiosities to serious therapeutic candidates. Importantly, ongoing clinical trials and development programs (summarized in Table 3) will determine how far this potential can be realized. These include Phase 3 trials for NPC (HPβCD); Phase 2 for Alzheimer’s (HPβCD); multiple Phase 1/2 trials in oncology (cyclodextrin–drug conjugates, siRNA delivery, etc.); and preclinical programs for atherosclerosis, kidney disease, and others [170].

**Table 3:** Translational status of cyclodextrin-based therapies and delivery systems. Overview of selected cyclodextrin-related interventions in various stages of development, from preclinical to approved. Abbreviations: IND = investigational new drug; Ph = phase; IV = intravenous; IT = intrathecal.

Cyclodextrin Therapy or Platform	Indication/Use	Status (Preclinical → Clinical)	Development Notes (Sponsor, Trial IDs, etc.)
HPβCD (VTS-270)	Niemann–Pick Type C (neurologic)	Phase 3 completed (results pending/regulatory)	Sponsor: Mallinckrodt (previously Vtesse/NIH); Intrathecal administration. Phase 2/3 (Trial NCT02534844) showed reduced disease progression; FDA considering approval [171].
HPβCD (Trappsol^®^ Cyclo™)	Niemann–Pick Type C (systemic)/Alzheimer’s disease	Phase 3 (NPC); Phase 2 (Alzheimer’s)	Sponsor: Cyclo Therapeutics. IV infusion. NPC IV trial (NCT02939547) ongoing; interim data show safety and some efficacy in organ function. Alzheimer’s trial (Phase 2, NCT05607615) recruiting to test cognitive endpoints. Fast Track granted for AD by FDA [121,172].
Methyl-β-CD (subcutaneous in vivo; 5 mM in vitro)	Atherosclerosis (reduced plaque burden; anti-inflammatory/anti-pyroptotic effect)	Preclinical (ApoE−/− mice on HFD + VSMC ox-LDL model)	Reduced aortic plaque area and CD68+ cell infiltration; improved lipid profile (LDL-c ↓, HDL-c ↑) and lowered IL-1β/IL-18; inhibited TLR4/NF-κB/NLRP3 signaling and GSDMD cleavage (GSDMD-NT ↓), consistent with reduced inflammasome-driven pyroptosis; no human trials reported [173].
Cyclodextrin dimer (VAR 200)	FSGS (focal segmental glomerulosclerosis); diabetic kidney disease	Preclinical (IND-enabling)	Sponsor: ZyVersa Therapeutics. Engineered CD dimer that sequesters cholesterol/7-ketocholesterol. Orphan designation for FSGS. Planning Phase 1 [174].
Sugammadex (Bridion^®^) (γ-CD derivative)	Reversal of neuromuscular blockade (anesthesia)	Approved (FDA 2015, EMA 2008)	First CD-based drug approved. Binds rocuronium and vecuronium. Not directly related to RCD, but proof of CD drug safety/efficacy (IV bolus) [175].
Sulfobutylether-β-CD (Captisol^®^)	Excipient for IV drug formulations (e.g., voriconazole, amiodarone, melphalan)	Approved (multiple NDAs)	Widely used solubilizer in >10 FDA-approved injectables. Generally safe but requires renal clearance. Its presence in formulations allowed drugs to reach market (e.g., IV voriconazole for fungal infections) [176].
CRLX101 (Cyclodextrin-Polymer Camptothecin)	Solid tumors (refractory cancers: renal, ovarian, SCLC)	Phase 2 (completed)	Developed by Cerulean Pharma. Phase 2 trials as monotherapy and with bevacizumab in renal cell and ovarian cancer showed modest activity. Company shifted focus; now being explored as Epothelione (EP0057) with PARP inhibitor in Phase 1b (NCT03386942) [103,177].
CALAA-01 (Cyclodextrin-siRNA nanoparticle)	Solid tumors	Phase 1 (completed)	First clinical demonstration of RNAi in humans, with tumor-localized nanoparticles and sequence-specific mRNA knockdown [178].
β-CD–siRNA nanoparticles	Cancer	Preclinical/Phase 1	Clinically validated tumor delivery and mRNA knockdown; multiple preclinical CD-polymer carriers for in vivo gene silencing [178,179].
HPβCD/βCD/γ-CD–drug inclusion complexes	Broad range of preclinical cancer models	Preclinical	Thymoquinone complex improved tumor suppression in mice [141]. Betulinic acid complexed with γ-CD showed enhanced bioavailability and significantly reduced tumor growth in a murine melanoma model [180]. Curcumin/HPβCD shown to increase bioavailability and apoptosis in tumor models [181]. The β-CD inclusion complex of phenoxodiol increased antiproliferative activity in neuroblastoma cells while reducing toxicity toward normal cells [182]. Albendazole complexed with sulfobutylether-β-CD significantly decreased malignant ascites in an ovarian cancer mouse model [183]. Caffeic acid phenethyl ester incorporated into γ-CD displayed greater stability, enhanced cytotoxicity, and improved in vivo tumor growth inhibition [184].
Acetalated-β-CD DHA nanoparticle	Cancer (solid tumors, erroptosis induction)	Preclinical (in vivo antitumor efficacy)	Acid-responsive nanocarrier enables tumor-targeted DHA delivery, promotes lipid peroxidation and ferroptosis, and significantly suppresses tumor growth in mouse models; no clinical studies reported [185].
β-CD polymer–RNA nanoparticle	Inflammation/cancer (gene silencing)	Preclinical → Phase 1 (CALAA-01 platform)	Clinically validated nucleic-acid delivery system; miR-223 is a known anti-inflammatory regulator in sepsis, but CD-based miR-223 therapy itself has not yet reached clinical studies [178,186].
Nasal β-cyclodextrin (intranasal spray)	Prevention of viral respiratory infections (physical barrier at nasal mucosa)	Preclinical—formulation development & in vitro (3D human nasal epithelium) + nasal cast deposition model	Forms a mucoadhesive in situ gel at 37 °C; non-cytotoxic and does not impair mucociliary beating; droplet size > 120 µm ensuring nasal (not lung) deposition; preferential turbinate coverage → prolonged residence time and pathogen-entry barrier [187].
Cyclodextrin-antitoxin therapy	Antidote for pore-forming toxins (e.g., pneumolysin in pneumonia)	Preclinical	Several patents on CD inhibitors of toxins. One example: β-CD oligomer that binds Clostridium perfringens α-toxin in rabbit model reduced hemolysis and mortality. Not yet in clinical development, but a promising adjunct to antibiotics in toxin-mediated infections [188,189,190].
Topical cyclodextrin formulations	Dermatology (acne—CD/retinoid); Wound healing (CD/antiseptic)	Approved (some cosmetics/derm)/late preclinical	Cyclodextrins used in acne gel (to solubilize retinoids, reduce irritation). Wound hydrogel with β-CD–iodine complex provides sustained antiseptic and may modulate inflammation (in trials). These leverage CDs to improve drug delivery and reduce cytotoxicity in skin cells (no direct RCD targeting, mainly formulation) [191].
Folinato-β-CD (FA-CD) targeting	Cancer (targeting folate receptor positive tumors like ovarian, AML)	Preclinical (in vitro)	FA-functionalization increased cellular uptake/delivery of payload in cancer cell lines (notably H460, Du-145), while cytotoxicity gains were not consistently improved; internalization occurs via multiple endocytosis pathways [192,193].

## 5. Methodology

A structured narrative (scoping) literature review was conducted to identify, critically appraise, and synthesize studies addressing the role of cyclodextrins in the modulation of regulated cell death pathways and their implications for immunometabolism and therapeutic applications. Literature identification followed PRISMA-ScR–informed reporting to ensure transparent documentation of search and selection processes. The primary databases consulted included PubMed, Web of Science, ScienceDirect, and Google Scholar. The search strategy prioritized articles published within the last five years to ensure inclusion of the most recent mechanistic insights and translational developments; however, seminal and foundational studies published earlier were also included when they provided essential mechanistic, experimental, or clinical context.

Search terms were constructed using combinations of keywords related to cyclodextrins and regulated cell death, including “cyclodextrins,” “β-cyclodextrin,” “hydroxypropyl-β-cyclodextrin,” “methyl-β-cyclodextrin,” “cholesterol modulation,” and “lipid rafts,” in conjunction with terms describing specific regulated cell death modalities such as “apoptosis,” “autophagy,” “autophagy-dependent cell death,” “pyroptosis,” “ferroptosis,” and “necroptosis”. Additional terms related to immune and metabolic regulation—such as “immunometabolism,” “macrophage polarization,” “inflammasome,” “NLRP3,” “TFEB,” “AMPK,” and “oxidative stress”—were incorporated to capture studies exploring immune cell reprogramming and metabolic signaling. For translational relevance, searches also included combinations with disease-related terms, including “cancer,” “atherosclerosis,” “cardiovascular disease,” “neurodegeneration,” “Niemann–Pick disease,” “Alzheimer’s disease,” “inflammatory disease,” and “lysosomal storage disorders”.

Inclusion criteria encompassed peer-reviewed original research articles and review papers that addressed at least one of the following aspects: (i) molecular or cellular mechanisms by which cyclodextrins influence regulated cell death pathways; (ii) the impact of cyclodextrin-mediated cholesterol or lipid modulation on immune cell function and immunometabolism; (iii) preclinical or clinical evidence linking cyclodextrin treatment to disease outcomes via modulation of cell death; or (iv) development and application of cyclodextrin-based delivery systems with relevance to apoptosis, autophagy, pyroptosis, ferroptosis, or necroptosis. Experimental studies conducted in vitro, in vivo animal models, and clinical or translational investigations were all considered, provided they offered mechanistic or therapeutic insights.

Exclusion criteria included non-peer-reviewed articles; conference abstracts without full data; editorials; case reports with fewer than ten subjects; non-English publications; and studies lacking direct relevance to regulated cell death, immunometabolic processes, or therapeutic applications of cyclodextrins. Articles focusing solely on the pharmaceutical excipient role of cyclodextrins without discussion of biological activity or cellular effects were also excluded.

**Figure 4:**
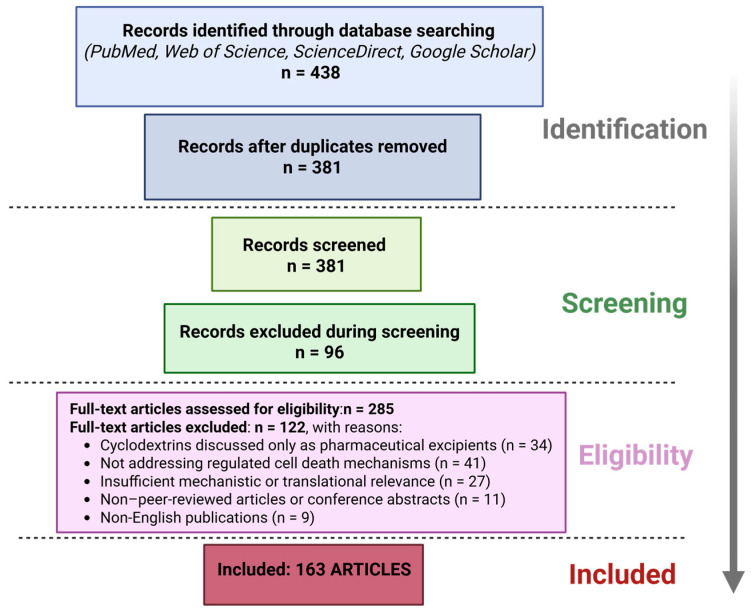
PRISMA-ScR–informed flow diagram illustrating the study identification, screening, and inclusion process. Created in BioRender. Varut, M. (2026) https://BioRender.com/071ce7p (accessed on 11 January 2026).

The initial screening was performed based on titles and abstracts retrieved from Google Scholar and PubMed, followed by full-text evaluation of potentially relevant studies obtained from PubMed, ScienceDirect, and publisher websites. Reference lists of selected articles were manually screened to identify additional relevant publications not captured in the primary search. The study selection process, including identification, screening, eligibility assessment, and final inclusion, is summarized in a PRISMA flow diagram (Figure 4).

## 6. Safety, Dose Dependency, and Translational Limitations

Although cyclodextrins are widely regarded as safe pharmaceutical excipients, their repurposing as bioactive modulators of regulated cell death and immunometabolism raises important safety and translational considerations. Accumulating evidence indicates that many of the biological effects underlying therapeutic efficacy are intrinsically dose-dependent and may become detrimental beyond a narrow therapeutic window. At low to moderate concentrations, cyclodextrins such as HPβCD can restore lipid homeostasis, activate TFEB-dependent lysosomal pathways, and promote protective autophagy. In contrast, excessive or prolonged exposure may lead to non-selective sterol extraction from cellular and organellar membranes, resulting in membrane destabilization, calcium dysregulation, impaired autophagic flux, and apoptosis. Such effects have been documented in hepatocytes, immune cells, and neurons, highlighting the risk of off-target cytotoxicity when dosing is not carefully controlled [24,70,103].

Ototoxicity represents one of the most clinically relevant adverse effects associated with high-dose or long-term HPβCD administration, particularly in the context of Niemann–Pick type C disease. Damage to cochlear outer hair cells has been attributed to cholesterol depletion in membranes essential for mechanotransduction, underscoring the vulnerability of sensory and neuronal tissues to cyclodextrin-induced sterol imbalance. In addition, excessive lysosomal cholesterol mobilization may overwhelm lysosomal capacity, leading to autophagic dysfunction rather than restoration, especially in metabolically active tissues [169,171,172].

Importantly, cyclodextrin safety is strongly influenced by chemical modification and formulation context. Methylated derivatives exhibit higher cholesterol affinity but increased cytotoxicity, whereas hydroxypropylated cyclodextrins generally display improved tolerability with reduced sterol-binding potency. Polymeric and nanoparticulate cyclodextrin systems introduce further formulation-dependent variables, including biodistribution, cellular uptake mechanisms, and degradation-related toxicity, which must be evaluated on a case-by-case basis [32,102,122,177].

Finally, significant interspecies differences in lipid metabolism, tissue sensitivity, and lysosomal function complicate the translation of preclinical safety data to humans. Doses tolerated in rodent models may elicit adverse effects in larger mammals or patients, emphasizing the need for cautious dose escalation, optimized delivery routes, and long-term safety monitoring [32,33,54]. Collectively, these limitations highlight that successful clinical translation of cyclodextrin-based therapies will depend on precise control of dose, formulation, and tissue targeting to balance therapeutic modulation of regulated cell death with acceptable safety profiles.

## 7. Conclusions

Cyclodextrins have evolved from inert pharmaceutical excipients to active modulators of cellular pathways, especially pathways governing regulated cell death and immune cell function. Their unique ability to bind lipids and alter membrane and lysosomal biology underpins this evolution. In this review, we highlighted how cyclodextrins can influence apoptosis, autophagy, pyroptosis, ferroptosis, and necroptosis—the major forms of regulated cell death—through mechanisms ranging from cholesterol depletion and membrane raft disruption to activation of autophagic clearance and modulation of inflammatory signaling. These mechanistic insights have been translated into diverse disease models, illustrating a unifying theme: by rebalancing cellular lipid metabolism and signaling, cyclodextrins often tip the scales away from pathological cell death.

In cancer, cyclodextrins can break therapy resistance and induce tumor cell death, either directly or as smart carriers for chemo- and ferroptosis-inducing agents. In cardiovascular disease, they can regress plaques by halting foam cell pyroptosis and fostering resolution of inflammation. In neurodegenerative and lysosomal storage disorders, cyclodextrins rescue cells from toxic lipid accumulation, staving off cell death and dysfunction. In inflammatory and infectious diseases, they emerge as immunomodulators that prevent excessive inflammatory cell death like pyroptosis or tune macrophage polarization to a healing state. Immunometabolic reprogramming of macrophages by cyclodextrins is a particularly exciting paradigm, connecting cholesterol efflux to suppression of inflammasomes and promotion of tissue repair.

The therapeutic innovation around cyclodextrins is accelerating. Clinical trials of HPβCD in NPC have demonstrated the feasibility of treating a CNS disease by clearing lysosomal lipids—something once thought unattainable. Trials in Alzheimer’s disease will test whether that paradigm extends to more common dementias. Cyclodextrin-based nanomedicines in oncology (for siRNA, chemotherapeutics, and immunotherapies) are refining the precision of cancer treatment. Even in fields like vaccinology or gene therapy, cyclodextrin polymers are being considered as delivery scaffolds.

Nevertheless, challenges remain. Cyclodextrins are non-selective in their extraction of lipids; achieving targeted delivery is crucial to avoid side effects such as off-target cytotoxicity or interference with normal cell function (e.g., hearing loss due to cholesterol removal in cochlea). Long-term safety data will be needed, as some cyclodextrins can accumulate (certain chemically modified CDs are not fully metabolized and rely on renal clearance). Immune reactions to larger CD constructs or impurities need monitoring. Dosing regimens must balance efficacy with toxicity, especially for chronic use. The cost of manufacturing high-quality cyclodextrin derivatives at scale and the complexities of regulatory approval for what are often combination products (drug + device-like carrier) also present hurdles.

## Figures and Tables

**Table 1:** Cyclodextrin types and modulation of regulated cell death pathways. Summary of various cyclodextrins (native and modified) and their demonstrated effects on major regulated cell death modalities. Abbreviations: MβCD = methyl-β-cyclodextrin; HPβCD = 2-hydroxypropyl-β-cyclodextrin; HPγCD = 2-hydroxypropyl-γ-cyclodextrin; DMβCD = 2,6-di-*O*-methyl-β-cyclodextrin; SBEβCD = Sulfobutylether-β-CD; CD = cyclodextrin; ROS = reactive oxygen species; TFEB = transcription factor EB; AMPK = AMP-activated protein kinase; ↑ = increase/activation; ↓ = decrease/inhibition.

Cyclodextrin (Type/Derivative)	RCD Pathway(s) Affected	Mechanism of Action	Examples
MβCD	Apoptosis (↑)	Induces mitochondrial cholesterol depletion, leading to alterations in mitochondrial structure and bioenergetics that favor apoptotic cell death [24].	Sensitized CML cells to apoptosis and, at higher concentrations, directly induced programmed cell death through downregulation of ERK/SPHK1 signaling [25].
	Pyroptosis (↓)	Depletes membrane cholesterol and disrupts lipid-raft-dependent inflammatory signaling upstream of inflammasome activation [26].	Reduces membrane cholesterol-dependent inflammatory signaling upstream of NLRP3 activation, a central pathway in macrophage pyroptosis and cytokine release in atherosclerosis [26,27].
	Ferroptosis (context-dependent)	Increases membrane fluidity by cholesterol removal, potentially promoting lipid peroxidation (↑ ferroptosis susceptibility); also used to solubilize sterols for ferroptosis studies [7,28].	Used in vitro to modulate cholesterol levels when studying ferroptosis suppression by cholesterol [29].
	Necroptosis (↓, indirect)	Disrupts cholesterol-rich membrane microdomains required for TNFR1 signaling and death complex assembly	Suggested to reduce TNF-induced necroptosis in some cell studies; contributes to NPC neuroprotection combined with RIPK1 inhibitors [7,30].
HPβCD	Apoptosis (↑ at high dose)	At high concentrations, sequesters cholesterol from organelle membranes, causing ER stress and caspase-8 activation; can trigger intrinsic apoptosis in cancer cells.	Induced G_2_/M arrest and apoptosis in leukemia cells, inhibiting proliferation and prolonging survival in vivo [31].
	Autophagy (↑ at mod. dose)	Activates TFEB and AMPK, enhancing autophagosome formation and lysosomal function. Helps clear accumulated substrates.	Reactivated autophagy in NPC1-deficient cells, reducing cholesterol storage promoted autophagy in vascular cells to prevent aneurysm [32].
	Autophagy (flux blockade at high dose)	Excessive cholesterol extraction destabilizes lysosomes, causing autophagosome accumulation (increased LC3-II, p62) and impaired autophagolysosome fusion.	20 mM HPβCD in hepatocytes blocked autophagic flux and led to apoptosis [33].
	Pyroptosis (↓)	Mobilizes cholesterol crystal deposits and dampens TLR4/NLRP3 signaling in macrophages (similar to MβCD)	Weekly HPβCD in LDLr^−^/^−^ mice reduced IL-1β and plaque inflammation. In vitro, lowered macrophage IL-1β release and pyroptotic death [34].
	Ferroptosis (↓ in normal cells, ↑ in tumor)	Cholesterol-depletion-mediated membrane remodeling alters susceptibility to lipid peroxidation; cyclodextrin inclusion complexes enhance the delivery of lipophilic redox-active anticancer agents.	Inclusion complexes improve the solubility and cellular availability of highly hydrophobic chemotherapeutics such as paclitaxel, illustrating the ability of cyclodextrins to enhance intracellular delivery of drugs functionally linked to oxidative lipid damage pathways. [6,35].
	Necroptosis (↓, indirect)	Alleviates underlying metabolic stress that triggers necroptosis; reduces neuroinflammation linked to necroptosis.	Combined with RIPK1 inhibition to extend survival in NPC disease; in diabetic kidney, reduced lipid-driven cell death [36].
HPγCD	Autophagy (↑)	Similar to HPβCD: activates TFEB, enhances lysosome–ER contact for lipid trafficking. Lower cholesterol affinity but impacts cellular pathways.	Overcame NPC1 deficiency by boosting autophagic clearance of cholesterol; increased lysosome-ER tethering [37].
DMβCD	Pyroptosis/Inflammation (↓)	Enhances solubility/bioavailability of progesterone via host–guest complexation; at high concentrations may disrupt lipid rafts through cholesterol extraction	PRO-entrapping DMβCD nanoparticles suppressed pCTS-L–driven cytokine/chemokine responses in peripheral blood mononuclear cells and improved survival in CLP-induced experimental sepsis with reduced inflammatory biomarkers [38].
Acetalated β-cyclodextrin (Ac-β-CD) (nanoparticle)	Ferroptosis (↑)	pH-degradable CD polymer that co-delivers pro-oxidants and iron; degrades in tumor acidity releasing ROS and iron to trigger ferroptotic death.	Nanoparticle with DHA and Fe^2+^/Fe^3+^ induced ferroptosis and tumor suppression in mice [39].
Cyclodextrin polymer (β-CD-polyamine)	Apoptosis (↑ via gene therapy)	Forms complexes with siRNA/anti-miRs to silence survival genes or reprogram cells. Induces apoptosis indirectly by gene knockdown.	β-CD polymer delivering anti-miR-33 to macrophages promoted cholesterol efflux and apoptosis of foam cells (reducing plaque cells) [40].
Folate-appended MβCD	Autophagy-dependent cell death (↑)	Selective uptake in FR-α-positive cancer cells via folate-receptor-dependent CLIC/GEEC endocytosis; induces autophagosome formation and autophagic flux; autophagy inhibitors (chloroquine, bafilomycin A1, 3-MA, LY294002) reduce cytotoxicity; associated with mitochondrial stress and mitophagy.	Induced autophagic vacuole formation in FR-α–expressing tumor cells (KB, M213); inhibition of autophagy attenuated cytotoxicity; no significant DNA fragmentation or caspase-3 activation (apoptosis-independent) [41].
SBEβCD (Captisol)	–	(Primarily an excipient, limited direct bioactivity; high doses can extract cholesterol).	Used to solubilize IV drugs (e.g., voriconazole); at high doses caused reversible kidney and liver phospholipidosis in animals (monitoring needed) [42].

**Table 2:** Cyclodextrin–disease links via regulated cell death modulation. Examples of how cyclodextrin (CD) interventions affect disease outcomes by targeting specific RCD pathways. Abbreviations: CD = cyclodextrin; RCD = regulated cell death; NPC = Niemann–Pick type C; AD = Alzheimer’s disease; RA = rheumatoid arthritis; MPS = mucopolysaccharidosis; FSGS = focal segmental glomerulosclerosis; TAM = tumor-associated macrophage; miR = microRNA.

Disease/Condition	Pathological RCD Involved	Cyclodextrin-Based Approach	Effect on Disease via RCD Modulation
Atherosclerosis	Macrophage pyroptosis; apoptosis of smooth muscle cells; secondary necrosis in plaques	MβCD or HPβCD (systemic administration); CD-loaded nanobubbles for cholesterol removal	Promotes cholesterol efflux and prevents cholesterol mobilization and LXR-dependent transcriptional reprogramming in macrophages, enhancing cholesterol efflux and reverse cholesterol transport, reducing cholesterol crystal burden and plaque inflammation, and thereby driving atherosclerosis regression [109,131].
Myocardial infarction (post-MI remodeling)	Cardiac dysfunction and profibrotic remodeling	Angiotensin-(1–7)/HPβCD inclusion complex (preclinical, oral, post-MI)	Improves left ventricular systolic function and myocardial deformation parameters and down-regulates profibrotic signaling (↓ TGF-β, ↓ collagen I), attenuating adverse post-infarction remodeling [132].
Abdominal aortic aneurysm	Vascular smooth muscle cell apoptosis and inflammatory wall remodeling HPβCD	HPβCD (preclinical, SC or IV)	Activates TFEB-dependent autophagy in VSMCs, reduces VSMC apoptosis and vascular inflammation, leading to decreased incidence and size of aneurysms in mice [133].
Diabetic kidney disease/FSGS	Podocyte lipid accumulation and injury; macrophage-driven glomerular inflammation	HPβCD (IV; VAR-200, preclinical/clinical development)	Mobilizes cholesterol and other lipids from podocytes and renal macrophages → reduces lipid-induced cellular stress, inflammation, and glomerulosclerosis; preserves podocyte structure and improves renal function in experimental models and early clinical studies [118,134].
Niemann–Pick type C disease	Neuronal lysosomal cholesterol accumulation and progressive neurodegeneration; microglial activation	HPβCD (intrathecal or IV; VTS-270, Trappsol)	Mobilizes lysosomal cholesterol in neurons and multiple organs → delays neurodegeneration and markedly prolongs survival in NPC mice; intrathecal administration slows neurological disease progression in patients [120,135].
Alzheimer’s disease	Neuronal and synaptic loss; amyloid-driven neurotoxicity; microglial activation	HPβCD (systemic administration; preclinical, clinical development)	Enhances brain cholesterol turnover and lysosomal function → reduces amyloid-β deposition and improves cognitive performance in AD mouse models; associated with increased autophagy and microglial-mediated clearance of amyloid [136].
Parkinson’s disease	Dopaminergic neuron loss and α-synuclein aggregation; neuroinflammation	HPβCD (preclinical)	Enhances lysosomal function and cholesterol trafficking → promotes clearance of α-synuclein aggregates and improves dopaminergic neuron survival and motor performance in PD models [137].
Huntington’s disease	Striatal neuron loss and mutant huntingtin aggregation; impaired autophagy and cholesterol homeostasis	HPβCD (preclinical)	Restores neuronal cholesterol turnover and enhances autophagy → reduces mutant huntingtin aggregates, improves motor performance and prolongs survival in HD mouse models [138].
Lysosomal lipase deficiency (Wolman, CESD)	Hepatocyte and macrophage injury due to lysosomal cholesteryl ester accumulation; hepatic inflammation and fibrosis	HPβCD (preclinical in mice)	Mobilizes lysosomal cholesterol in liver and reticuloendothelial cells → reduces hepatic lipid storage, inflammation, and fibrosis and prolongs survival in Lal^−^/^−^ mice [139].
Cancer	Evasion of apoptosis; therapy resistance; immunosuppressive tumor microenvironment	Cyclodextrins (MβCD; HPβCD inclusion complexes; CD-based nanocarriers)	(1) Cholesterol depletion disrupts lipid rafts → resensitizes tumor cells to apoptosis and enhances uptake and efficacy of chemotherapeutics [140]. (2) Inclusion complexes improve solubility and bioavailability of hydrophobic drugs (e.g., thymoquinone, paclitaxel) → increased cancer cell death. [141,142]. (3) CD-based nanocarriers enable functional siRNA delivery (including across BBB models), achieving intracellular release and target gene silencing [143].
Breast cancer (specific)	Drug resistance (apoptosis avoidance via Akt/ERK); cancer stem cell survival	MβCD + chemotherapeutics/HPβCD	MβCD co-treatment lowered membrane cholesterol → ↓ caveolin-1 and ↓ pAkt, pERK, thereby enhancing chemotherapy-induced apoptosis of breast cancer cells [140]. HPβCD (monotherapy) → up to 100% tumor regression in early-stage TNBC xenografts; strong reduction in intermediate/late tumors with increased apoptosis and no liver toxicity [31]; HPβCD inclusion complex with palbociclib → markedly increased aqueous solubility, cellular uptake, apoptosis and cytotoxicity in MDA-MB-231 breast cancer cells (in vitro) [144].
Lung cancer (NSCLC)	Chemo resistance; need for new cell death induction (ferroptosis)	HPβCD–thymoquinone inclusion; CD-based ROS-ferroptosis nanomedicine	HPβCD–TQ complex increased TQ solubility and delivery → induced ferroptosis in NSCLC via NF-κB-mediated pathway, inhibiting tumor growth [141]. Acetalated β-CD nanoparticle delivering dihydroartemisinin and Fe^2+^ generates ROS in the acidic tumor microenvironment, induces ferroptosis, and exhibits a synergistic antitumor effect when combined with chemotherapy [145].
Leukemia (AML, CML)	Leukemic cell survival, including drug-resistant clones; high cholesterol in blasts	HPβCD (IV or IP preclinical); FA-HPβCD (targeted)	HPβCD monotherapy: triggers apoptosis in AML/CML blasts (including TKI-resistant) by cholesterol removal and G_2/M arrest → prolonged survival in mouse models. Folate-HPβCD: targeted uptake in leukemia cells → induces autophagic cell death and apoptosis. Minimal toxicity to normal cells observed at therapeutic doses [146].
Tumor immunotherapy (TME modulation)	Tumor-associated macrophages (TAMs) sustaining immunosuppression; poor antigen presentation (dendritic cell dysfunction)	β-CD nanoparticles delivering miR-125b inhibitors or anti-miR-33 to TAMs; CD conjugates with STING agonists for dendritic cells	Anti-miR33 CD-nanotherapy in TAMs → ↑ cholesterol efflux, drives M2→M1 repolarization, enhances IL-12 and antigen presentation, thus boosting anti-tumor T-cell responses. CD-STING conjugates: improve solubility and targeting of STING agonists, leading to localized tumor cell pyroptosis and immune activation (under investigation) [147,148].
Rheumatoid arthritis (RA)	Synovial inflammation driven by macrophage-mediated pro-inflammatory cytokine production with NLRP3 inflammasome involvement and a persistent inflammatory microenvironment	Amphiphilic β-cyclodextrin nanoparticles (CD-NPs: CDOC6, CDOC12, CDSC6) compared with soluble β-cyclodextrin, representing a drug-free, biomaterial-intrinsic immunomodulatory strategy targeting macrophages	Cyclodextrin nanoparticles (CDOC6, CDOC12, CDSC6) attenuate inflammation by suppressing pro-inflammatory cytokine production, downregulating costimulatory activation markers, reducing NLRP3 inflammasome activity, and reprogramming macrophages toward an anti-inflammatory phenotype [149].
Systemic sepsis (endotoxemia)	Exuberant macrophage pyroptosis and cytokine storm; lymphocyte apoptosis causing immunosuppression	DMβCD (injectable); β-CD–miR-223 NP (as immunomodulator)	DMβCD binds LPS and suppresses LPS-induced macrophage activation, reducing NO and TNF-α production and significantly improving survival in LPS/D-galactosamine-induced endotoxin shock in mice [150]. β-CD–miR-223 NP delivers miR-223 to macrophages, suppresses NF-κB signaling, promotes M2 polarization, and reduces TNF-α, IL-1β and IL-6 [91].
COVID-19/viral ARDS	Inflammasome activation in lungs; endothelial pyroptosis; viral entry via cholesterol-rich rafts	2,6-DMβCD and 3-*O*-ethyl-βCD (nebulized or IV); HPβCD	Cyclodextrin derivatives applied to respiratory epithelium can extract cholesterol from cell membranes and viral envelopes → inhibit SARS-CoV-2 entry and replication. Also reduce lung inflammasome activation by limiting virus-induced cholesterol crystal formation [151,152,153].
HIV infection	Need for effective local vaginal ARV delivery for prevention of sexual transmission	HPβCD-based mucoadhesive freeze-dried vaginal discs (with surfactants)	Cyclodextrin improves drug solubilization, modulates release, and enhances mechanical properties and mucoadhesion, enabling rapid on-demand vaginal delivery of tenofovir or dapivirine; in combination with sodium dodecyl sulfate, it alters gel microstructure and supports rapid local drug release after administration, making the system suitable for on-demand HIV prevention [154].
Bacterial infections (general)	Toxin-mediated pore formation and host-cell lysis	CD-based toxin inhibitors (β-CD derivatives)	β-CD derivatives neutralize pore-forming toxins (e.g., *S. aureus* α-hemolysin), blocking transmembrane channel formation and preventing toxin-induced cell death. In murine MRSA pneumonia models, treatment reduced epithelial injury and mortality [155].
Cryopyrinopathies	Constitutive NLRP3 inflammasome activation in myeloid cells → excessive IL-1β maturation and pyroptosis-driven systemic inflammation	HPβCD (experimental, cholesterol-mobilizing inflammasome modulator)	HPβCD enhances cholesterol solubilization and LXR signaling, which represses NF-κB- and NLRP3-dependent transcription and decreases IL-1β production; in NLRP3 gain-of-function mice, this was associated with reduced splenic IL-1β and improved weight gain, consistent with attenuation of inflammasome-driven inflammation [109,149,156].
Gout (arthritis caused by monosodium urate crystal deposition)	MSU crystals activate the NLRP3 inflammasome in macrophages → IL-1β release, neutrophil recruitment and pyroptosis-driven joint inflammation	HPβCD or hyperbranched CD polymers (intra-articular/systemic, experimental)	Cyclodextrins form inclusion complexes with uric acid, increasing its solubility, preventing MSU crystal formation, and mobilizing existing deposits. In a murine MSU-induced gout model, this resulted in reduced joint inflammation; lower IL-1β, IL-6, and TNF levels; and synergistic efficacy with standard anti-gout drugs [157].
Mucopolysaccharidoses (MPS I/II/III)	Accumulation of glycosaminoglycans with secondary storage of non-cholesterol lipids (e.g., gangliosides) contributing to cellular dysfunction in lysosomal storage disease	Cyclodextrins (HPβCD; experimental substrate-mobilizing agent)	Promotes reduction of secondary lipid accumulation in lysosomal storage disorders, including gangliosides, and targets pathological macromolecule storage (e.g., GAG-associated disease burden); proposed as adjunct to enzyme-based therapies to improve cellular homeostasis [158].

## Data Availability

No new data were created or analyzed in this study. Data sharing is not applicable.

## Abbreviations

The following abbreviations are used in this manuscript:

AAA	Abdominal aortic aneurysm
ABCA1	ATP-binding cassette transporter A1
ABCG1	ATP-binding cassette transporter G1
Ac-β-CD	Acetalated β-cyclodextrin
AD	Alzheimer’s disease
Akt	Protein kinase B
AML	Acute myeloid leukemia
AMPK	AMP-activated protein kinase
α-CD	Alpha-cyclodextrin
β-CD	Beta-cyclodextrin
BBB	Blood–brain barrier
Bcl-2	B-cell lymphoma 2
Bax	Bcl-2-associated X protein
CALAA-01	Cyclodextrin polymer-based siRNA nanoparticle
CAPTISOL	Sulfobutylether-β-cyclodextrin formulation
CD	Cyclodextrin
CML	Chronic myeloid leukemia
CNS	Central nervous system
CRLX101	Cyclodextrin–camptothecin nanoparticle
DAMPs	Damage-associated molecular patterns
DHA	Dihydroartemisinin
DMβCD	2,6-Di-*O*-methyl-β-cyclodextrin
DSPE-PEG	1,2-Distearoyl-sn-glycero-3-phosphoethanolamine–polyethylene glycol
EGFR	Epidermal growth factor receptor
ER	Endoplasmic reticulum
FA-HPβCD	Folate-appended hydroxypropyl-β-cyclodextrin
GSDMD	Gasdermin D
GPX4	Glutathione peroxidase 4
γ-CD	Gamma-cyclodextrin
HDL	High-density lipoprotein
HMGB1	High mobility group box 1
HPβCD	2-Hydroxypropyl-β-cyclodextrin
HPγCD	2-Hydroxypropyl-γ-cyclodextrin
IL	Interleukin
iNOS	Inducible nitric oxide synthase
LC3	Microtubule-associated protein 1 light chain 3
LPS	Lipopolysaccharide
LSDs	Lysosomal storage disorders
LXR	Liver X receptor
MAPKs	Mitogen-activated protein kinases
MβCD	Methyl-β-cyclodextrin
MCP-1	Monocyte chemoattractant protein-1
miR/miRNA	MicroRNA
MLKL	Mixed lineage kinase domain-like protein
M1	Classically activated macrophage phenotype
M2	Alternatively activated macrophage phenotype
NF-κB	Nuclear factor kappa B
NLRP3	NLR family pyrin domain containing 3
NOX	NADPH oxidase
NPC	Niemann–Pick disease type C
NPC1	Niemann–Pick disease type C1
NSCLC	Non-small cell lung cancer
PDPA	Poly(2-(diisopropylamino)ethyl methacrylate)
PI3K	Phosphoinositide 3-kinase
RCD	Regulated cell death
RIPK1	Receptor-interacting serine/threonine-protein kinase 1
RIPK3	Receptor-interacting serine/threonine-protein kinase 3
ROS	Reactive oxygen species
SBEβCD	Sulfobutylether-β-cyclodextrin
siRNA	Small interfering RNA
STAT3	Signal transducer and activator of transcription 3
TAM	Tumor-associated macrophage
TFEB	Transcription factor EB
TLR4	Toll-like receptor 4
TME	Tumor microenvironment
TNF	Tumor necrosis factor
TQ	Thymoquinone
TUNEL	Terminal deoxynucleotidyl transferase dUTP nick end labeling
